# Engineered Cas12a-Plus nuclease enables gene editing with enhanced activity and specificity

**DOI:** 10.1186/s12915-022-01296-1

**Published:** 2022-04-25

**Authors:** Hongxin Huang, Guanjie Huang, Zhihong Tan, Yongfei Hu, Lin Shan, Jiajian Zhou, Xin Zhang, Shufeng Ma, Weiqi Lv, Tao Huang, Yuchen Liu, Dong Wang, Xiaoyang Zhao, Ying Lin, Zhili Rong

**Affiliations:** 1 Dermatology Hospital, Southern Medical University, Guangzhou, 510091 China; 2 Cancer Research Institute, School of Basic Medical Sciences, State Key Laboratory of Organ Failure Research, National Clinical Research Center of Kidney Disease, Key Laboratory of Organ Failure Research (Ministry of Education), Southern Medical University, Guangzhou, 510515 China; 3 Department of Bioinformatics, School of Basic Medical Sciences, Southern Medical University, Guangzhou, 510515 China; 4 Department of Development, School of Basic Medical Sciences, Southern Medical University, Guangzhou, China; 5 Experimental Education/Administration Center, School of Basic Medical Sciences, Southern Medical University, Guangzhou, 510515 China

**Keywords:** Cas12a-Plus, High-activity and high-specificity, CRISPR, Engineering, Gene editing

## Abstract

**Background:**

The CRISPR-Cas12a (formerly Cpf1) system is a versatile gene-editing tool with properties distinct from the broadly used Cas9 system. Features such as recognition of T-rich protospacer-adjacent motif (PAM) and generation of sticky breaks, as well as amenability for multiplex editing in a single crRNA and lower off-target nuclease activity, broaden the targeting scope of available tools and enable more accurate genome editing. However, the widespread use of the nuclease for gene editing, especially in clinical applications, is hindered by insufficient activity and specificity despite previous efforts to improve the system. Currently reported Cas12a variants achieve high activity with a compromise of specificity. Here, we used structure-guided protein engineering to improve both editing efficiency and targeting accuracy of *Acidaminococcus* sp. Cas12a (*As*Cas12a) and *Lachnospiraceae bacterium* Cas12a (*Lb*Cas12a).

**Results:**

We created new *As*Cas12a variant termed “*As*Cas12a-Plus” with increased activity (1.5~2.0-fold improvement) and specificity (reducing off-targets from 29 to 23 and specificity index increased from 92% to 94% with 33 sgRNAs), and this property was retained in multiplex editing and transcriptional activation. When used to disrupt the oncogenic BRAF^V600E^ mutant, *As*Cas12a-Plus showed less off-target activity while maintaining comparable editing efficiency and BRAF^V600E^ cancer cell killing. By introducing the corresponding substitutions into *Lb*Cas12a, we also generated *Lb*Cas12a-Plus (activity improved ~1.1-fold and off-targets decreased from 20 to 12 while specificity index increased from 78% to 89% with 15 sgRNAs), suggesting this strategy may be generally applicable across Cas12a orthologs. We compared Cas12a-Plus, other variants described in this study, and the reported enCas12a-HF, enCas12a, and Cas12a-ultra, and found that Cas12a-Plus outperformed other variants with a good balance for enhanced activity and improved specificity.

**Conclusions:**

Our discoveries provide alternative *As*Cas12a and *Lb*Cas12a variants with high specificity and activity, which expand the gene-editing toolbox and can be more suitable for clinical applications.

**Supplementary Information:**

The online version contains supplementary material available at 10.1186/s12915-022-01296-1.

## Background

The clustered regularly interspaced short palindromic repeats (CRISPR)-CRISPR associated protein (Cas) system, an adaptive immunity system in bacteria and archaea, is a promising genome editing tool that has been widely used in a broad range of areas [1]. However, off-target cleavage of Cas-nucleases is routinely observed and remains an obstacle for clinical applications [2–4]. Therefore, the improvement of their targeting accuracy is essential for CRISPR-Cas tools in genome editing research, particularly in therapeutic applications [1]. So far, several strategies have been developed to improve this technique, and they can generally be divided into two categories: the sgRNA modification [5–16] and the Cas-nuclease protein engineering [17–27]. In particular, protein engineering is an efficient and widely used approach for the development of high-fidelity Cas-nuclease variants, which has been well-proved in *Sp*Cas9, such as the unbiased engineered variants HiFi-Cas9 [17], evoCas9 [18], xCas9 [19], Sniper-Cas9 [20], LZ3-Cas9 [21], etc., and the structure-guided engineered mutants *Sp*Cas9-HF [22], e*Sp*Cas9 [23], HeF*Sp*Cas9 [24], HypaCas9 [25], etc. These novel high-specific Cas-nuclease variants broaden the repertoire of CRISPR-Cas9 tools in gene, epigenome, and base editing applications [28].

*As*- (*Acidaminococcus sp.*) and *Lb*- (*Lachnospiraceae bacterium*) Cas12a are the two commonly used Cas-nuclease in the CRISPR-Cas12a system, which is also a promising genome editing tool in addition to the extensively investigated CRISPR-Cas9 [29, 30]. Several unique features make Cas12a distinguished from Cas9. First, Cas12a recognizes T-rich PAMs and generates sticky break ends [29], which makes it a complement to Cas9 in genome editing and broadens the genomic targeting scope. Second, Cas12a is a single crRNA-guided endonuclease and has the ribonuclease activity to process its pre-crRNA into mature crRNA [29, 31], which enables multiplex editing in a single crRNA transcript [32, 33]. Third, rather than using both RuvC and HNH domains in Cas9 [34], Cas12a cuts target DNA with a single RuvC domain. Fourth, Cas12a possesses the ability to trans-cleave single-stranded DNA (ssDNA) [35], making it a powerful platform for nucleic acid detection [35, 36]. Finally, Cas12a displays less off-target nuclease activity than does Cas9 [37, 38], enabling more precise genome editing for therapeutic applications [39].

Although Cas12a nuclease has shown powerful potentials in gene editing [40], insufficient efficiency and specificity remain a major obstacle for its broad application [37, 38]. Similar to *Sp*Cas9, approaches including Cas-protein engineering and sgRNA-modification had been employed in CRISPR-*As*Cas12a/*Lb*Cas12a system to improve their cutting efficiency or targeting accuracy [6–16, 26, 27, 41–53]. However, none of them achieved both improved specificity and enhanced activity (Additional file 1: Table S1) [6–16, 26, 27, 41–53], calling for the development of new variants to improve this system. Moreover, other Cas12a orthologs also have been explored and engineered for the same purpose (Additional file 1: Table S2) [42, 54–63], reflecting the urgent needs in this field. Here, we developed high-active and high-specific Cas12a nuclease variants by structure-guided protein engineering, which can expand the CRISPR-Cas toolbox and provide new genome editing tools for the applications in fundamental research and translational medicine.

## Results

### Structure-guided protein engineering for high-fidelity AsCas12a variants

**Fig. 1:**
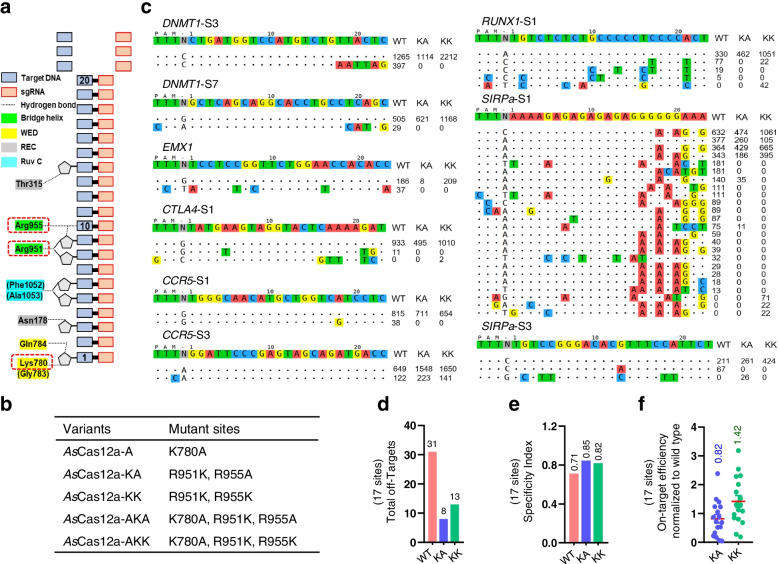
Generation of *As*Cas12a variants with increased specificity by weakening non-specific DNA contacts. **a** Schematic of wild-type *As*Cas12a interaction with the target DNA-sgRNA duplex. **b** The mutation sites of the *As*Cas12a variants. **c** Tag-seq-based comparative analyses of wild-type *As*Cas12a (WT), *As*Cas12a variant KA (KA), and *As*Cas12a variant KK (KK) with seventeen sgRNAs targeting nine genes (also see Additional file 2: Figure S1e). The sgRNA was shown on the top and the on-target and the off-target cleavages were displayed without or with mismatches to the sgRNA reference by color highlighting. Sequencing read counts were shown to the right of each site. **d** Total number of off-target sites detected with the seventeen sgRNAs. **e** Specificity Index (value was calculated by the ratio of total on-target reads to the on-target reads plus the off-target reads within the seventeen sites). **f** Normalization of on-target activity of KK and KA to wild-type *As*Cas12a

To generate the high-fidelity *As*Cas12a mutant, we employed the energy hypothesis [22, 23], because numerous high specific Cas-nuclease variants had been successfully developed based on this hypothesis, including Cas9-HF [22] and e*Sp*Cas9 [23]. According to the crystal structure of the *As*Cas12a-crRNA-target DNA complex [64], we identified three positively charged amino acid residues (K780, R951, and R955) forming hydrogen bond contact with the backbone of the target DNA strand (Fig. 1a). R951 and R955 are located in the Bridge helix and K780 is in the WED domain [64], all of which are likely to be involved in stabilizing the interaction between *As*Cas12a and the targeted strand and thus leading to cleave mismatched off-target sites [22, 23, 64]. We then mutated these three amino acids and combined them for constructing five different *As*Cas12a variants bearing single, double, or triple substitutions (Fig. 1b), and tested whether these mutants possessed a relatively higher specificity. Western blotting showed that these mutants were expressed equivalently to the wild-type (WT) *As*Cas12a (Additional file 2: Figure S1a). Then, we performed the editing assay by using mismatched sgRNAs targeting *DNMT1*-site3, since this site had been well-studied in wild-type *As*Cas12a with different mismatched crRNAs [38]. Using deep-sequencing, T7 endonuclease I (T7E1), and polyacrylamide gel electrophoresis (PAGE)-based methods, we found that the *As*Cas12a-KK and *As*Cas12a-KA mutants retained comparable on-target activities but fewer cleavages with mismatched sgRNAs (Additional file 2: Figure S1b-d), indicating they may have a higher specificity. Based on this, we chose *As*Cas12a-KK and *As*Cas12a-KA variants for further study.

### Genome-wide specificity of AsCas12a-KK and AsCas12a-KA

To globally evaluate the editing specificity of *As*Cas12a-KK and *As*Cas12a-KA, we performed Tag-seq experiments [65] to assess seventeen different sgRNAs targeting different sites in the endogenous human *EMX1*, *DNMT1*, *RUNX1*, *PD1*, *CTLA4*, *CD47*, *SIRPa*, *CCR5*, and *CXCR4* genes (Fig. 1c and Additional file 2: Figure S1e), as these sites had been well-studied or were of clinical relevance. As a result, Tag-seq showed that the off-target cleavage was significantly decreased for *As*Cas12a-KK and *As*Cas12a-KA variants at most of the tested sites, with reducing the total off-target sites from 31 with *As*Cas12a-WT to 13 with *As*Cas12a-KK and to 8 with *As*Cas12a-KA (Fig. 1d). As expected, the specificity of the two mutants was increased, with the specificity index of 0.85, 0.82, and 0.71 for KA, KK, and WT, respectively (Fig. 1e). However, *As*Cas12a-KA was less active with only an average of 82% editing efficiency compared to the WT *As*Cas12a (Fig. 1f).

### Improvement of the AsCas12a mutants for genome editing

**Fig. 2:**
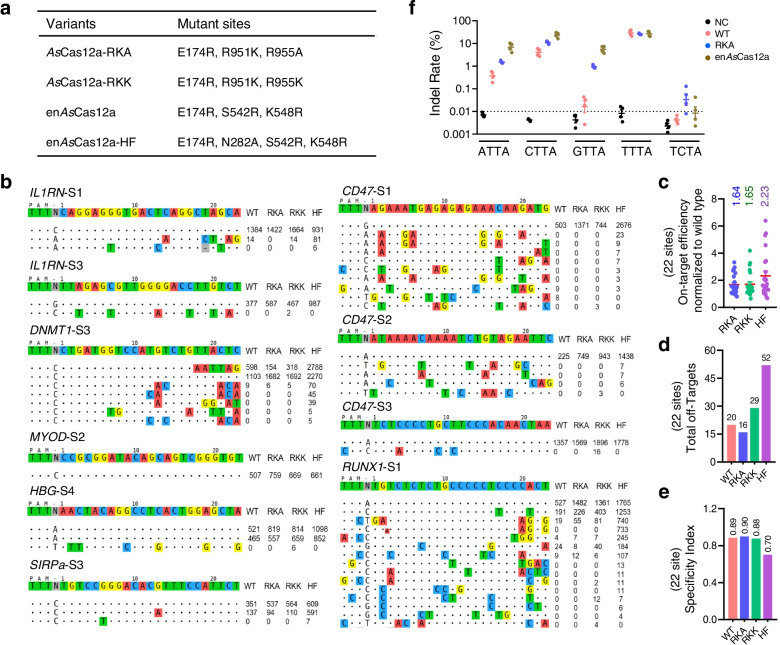
Generation of *As*Cas12a variants with increased efficiency by introducing the high-activity substitution. **a** The mutation sites of the high active *As*Cas12a variants. **b** Tag-seq-based comparative analyses of wild-type *As*Cas12a (WT), *As*Cas12a variant RKA (RKA), *As*Cas12a variant RKK (RKK), and *As*Cas12a-HF (HF, the reported high-fidelity variant of en*As*Cas12a) with twenty-two sgRNAs targeting twelve genes (also see Additional file 2: Figure S3). **c** Normalization of on-target activity of RKA, RKK, and HF to wild-type *As*Cas12a. **d** Total number of off-target sites detected with the twenty-two sgRNAs. **e** Specificity index (value was calculated by the ratio of total on-target reads to the on-target reads plus the off-target reads within the twenty-two sites). **f** Detection of the editing abilities for the non-canonical PAM with *As*Cas12a-RKA. Mean values are presented with SEM, *n*=4 independent experiments. Indel was revealed by Deep-seq

Inspired by the enhanced *As*Cas12a (en*As*Cas12a), a highly active *As*Cas12a mutant previously reported [27], we next constructed *As*Cas12a-RKA mutant by introducing the E174R substitution (Fig. 2a), because this site was proximal to PAM DNA [64] and the charged arginine residue mutation could alter or form novel PAM proximal DNA contacts, which had been proved to increase the editing activity of the *As*Cas12a nuclease [27]. Western blotting showed that this substitution did not affect protein expression (Additional file 2: Figure S2a). Then, we performed specificity comparative analyses among *As*Cas12a-WT, *As*Cas12a-RKA, and *As*Cas12a-HF (en*As*Cas12a-HF, the high-fidelity version of en*As*Cas12a) by targeting *RUNX1* and Site 6, two well-studied sites for specificity assessment of the CRISPR-Cas12a system [38]. Tag-seq indicated that the *As*Cas12a-RKA increased the editing efficiency with ~3-fold promotion (on-target reads was 2922 in RKA while WT is 1059) at site *RUNX1* and at least equivalent editing level at Site 6 (2054 reads in RKA versus 1951 in WT) (Additional file 2: Figure S2b, c). More importantly, unlike the HF mutant, the activity improvement of the RKA mutant did not compromise but slightly improved the specificity (Additional file 2: Figure S2b-e).

Next, to determine whether this strategy could be applied to another variant, KK, we constructed *As*Cas12a-RKK as well and examined the protein expression level (Fig. 2a and Additional file 2: Figure S2a). Then, we utilized Tag-seq with twenty-two sgRNAs targeting twelve genes to comprehensively assess the editing abilities among *As*Cas12a-WT, *As*Cas12a-RKA, *As*Cas12a-RKK, and *As*Cas12a-HF (Fig. 2b and Additional file 2: Figure S3). Expectedly, with the introduction of the E174R, the average efficiency of RKA was increased about 1.64-fold compared to the WT *As*Cas12a (Fig. 2c). Moreover, among the four tested enzymes, RKA exhibited the highest specificity with the least off-target sites (Fig. 2d, e). Notably, the improvement of activity was also observed in mutant RKK (Fig. 2c); however, its specificity was slightly affected (Fig. 2d, e). Consistent with the previous report [27], en*As*Cas12a-HF displayed a robust efficiency which was ~2.23-fold improvement compared to WT *As*Cas12a (Fig. 2c); however, it induced 32 additional off-target cleavages in twenty-two tested sgRNAs and exhibited the lowest specificity (Fig. 2d, e). These data demonstrated that with the combination of E174R substitution, *As*Cas12a-RKA exhibited improved activity and slightly increased specificity, indicating it was a high-active and high-specific Cas12a nuclease. As the high-active site E174R displayed extended targeting range for non-canonical PAMs (such as ATTA, CTTA, GTTA, and TCTA) [27], we next tested whether *As*Cas12a-RKA possessed this ability. As shown in Fig. 2f, *As*Cas12a-RKA induced indels to an extent between en*As*Cas12a and WT *As*Cas12a, indicating a slightly expanded PAM recognition.

Apart from protein engineering, sgRNA modification is also an efficient way of enhancing the efficiency of the CRISPR-Cas12a system [66]. It had been reported that adding a “U4AU6” motif at the end of the crRNA [6] or using a pol-II-driven truncated pre-tRNA [7] to express the crRNA could improve the activity of the CRISPR-Cas12a system (Additional file 2: Figure S4a). Thus, we designed such sgRNAs to target exogenous *EGFP* gene and endogenous genes, *FANCF* and Site 6. FACS and Deep-seq results showed that the U4AU6-crRNA combined with some Cas12a variants tended to increase the editing efficiency at *EGFP*-g1 and site 6 loci, while the truncated pre-tRNA displayed comparable or less efficiency at all the four tested sites (Additional file 2: Figure S4b, c), suggesting that these two methods may work in a site-dependent manner, similar to a previous report of improving *Sp*Cas9 specificity by truncated-sgRNA [2, 5].

### Multiplex editing of the AsCas12a variants using a single crRNA array

**Fig. 3:**
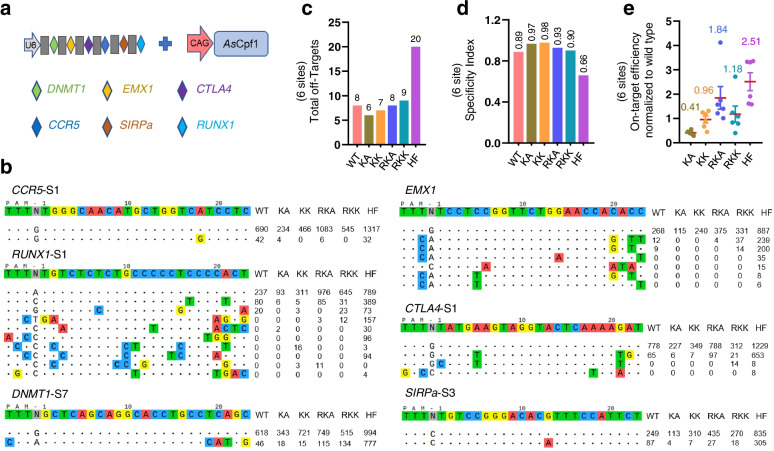
Multiplex editing with the *As*Cas12a variants. **a** Schematic of the multiplex editing. **b** Tag-seq-based comparative analyses of wild-type *As*Cas12a (WT), *As*Cas12a variants KA, KK, RKA, RKK, and HF with a single crRNA array targeting six sites. **c** Total number of off-target sites detected with the six sgRNAs within a single crRNA array. **d** Specificity index (value was calculated by the ratio of total on-target reads to the on-target reads plus the off-target reads counts within these six sites). **e** Normalization of on-target activity of KA, KK, RKA, RKK, and HF to wild-type *As*Cas12a in multiplex editing

Next, we tested whether the new *As*Cas12a variants could improve Cas12a-based approaches. An advantage of the Cas12a enzyme over Cas9 is the multiplex editing, in which Cas12a processes individual crRNAs from a single crRNA array to simplify multiplex targeting in cells [32, 33, 47]. To assess this property in the engineered Cas12a variants, we cloned a poly-crRNA transcript including six crRNAs targeting *DNMT1*, *EMX1*, *CTLA4*, *CCR5*, *SIPRa*, and *RUNX1* (Fig. 3a). Tag-seq experiments showed that all the mutants could mediate gene editing in these six sites with different levels (Fig. 3b), demonstrating the amino acid substitutions did not affect the crRNA self-processing activity. Among all the tested variants, RKA displayed both improved efficiency and specificity, while the en*As*Cas12a-HF showed the highest activity but with the worst specificity (Fig. 3b–e). These results demonstrated that *As*Cas12a-RKA was able to do multiplex editing with high activity and slightly improved specificity.

### Transcriptional activation with the AsCas12a variants

**Fig. 4:**
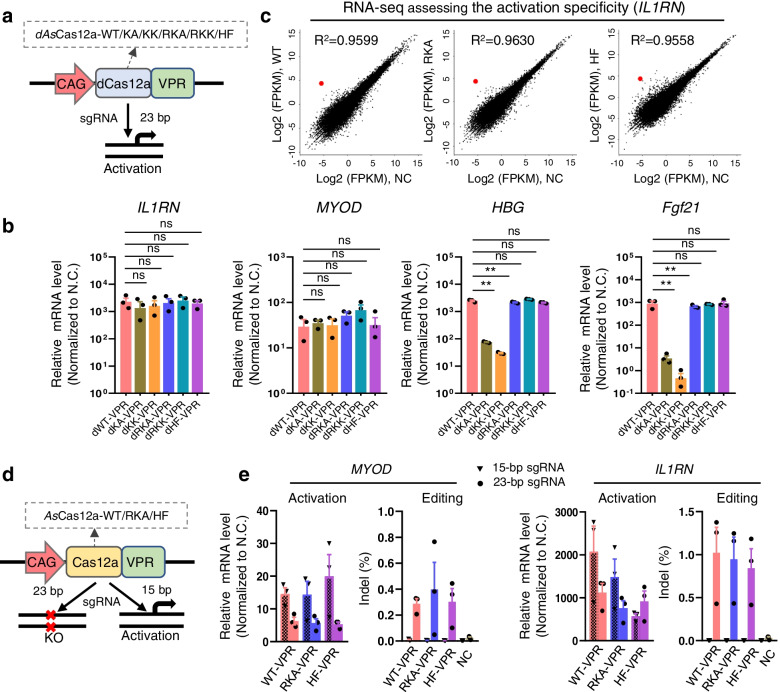
Transcriptional activation with the *As*Cas12a variants. **a** Schematic of the gene activation system based on catalytic-dead *As*Cas12a variants. VPR, synthetic VP64-p65-Rta activation domain. **b** qPCR analyses of the transcriptional activation levels with the *As*Cas12a variants guided by a single sgRNA targeting each promoter region of *IL1RN*, *MYOD*, and *HBG* in human HEK293T cells and *Fgf21* in mouse B16 cells, respectively. Mean values are presented with SEM, *n*=3 independent experiments. **p<0.01. ns, no significance. Student’s *t*-test, dWT-VPR sample versus corresponding d*As*Cas12a-VPR variant samples. **c** RNA-seq assessing the specificity of the dWT-, dRKA-, and dHF-VPR systems to activate *IL1RN* gene. **d** Schematic of the orthogonal gene editing and activation system based on catalytic active *As*Cas12a variants. **e** Relative mRNA expression (Activation) and genome editing efficiency (Editing) of the *As*Cas12a-VPR systems at *MYOD* and *IL1RN* sites with indicated lengthed sgRNAs. 15-bp sgRNAs for gene activation and 23-bp sgRNAs for genome editing. Mean values are presented with SEM, *n*=3 independent experiments. Indel was revealed by Deep-seq

Cas12a has also been used for transcriptional activation of endogenous genes by fusing DNase-inactive Cas12a (dCas12a) to a gene activator [67, 68]. We then examined this application with the engineered *As*Cas12a variants. We found that using d*As*Cas12a fused to the synthetic VPR (VP64-p65-Rta) activation domain (d*As*Cas12a-VPR) (Fig. 4a), the dRKA-, dRKK-, and dHF-VPR systems can activate the transcriptional expression of *IL1RN*, *MOYD*, and *HBG* in human cells and *Fgf21* in mouse cells with comparable level to dWT-VPR (Fig. 4b). However, dKK-VPR and dKA-VPR showed much lower capability to activate *HBG* and even failed to activate *Fgf21* (Fig. 4b), which might reflect that the binding ability at these two sites was remarkably attenuated [64, 69]. Next, we performed RNA-seq for specificity comparison among dWT-, dRKA-, and dHF-VPR to activate *IL1RN*. As a result, the dRKA-VPR system displayed a slight improvement in activation of the endogenous gene *IL1RN* compared with the WT and HF (Fig. 4c), demonstrating the ability of the RKA in transcriptional activation.

Simultaneous orthogonal gene activation and genome editing for multiplex genes with catalytical active *Sp*Cas9 or *As*Cas12a fused to a gene activator has been reported [32, 70, 71]. Therefore, we compared orthogonally activation and editing ability among catalytically active *As*Cas12a-WT, *As*Cas12a-RKA, and *As*Cas12a-HF fused to VPR (termed WT-VPR, RKA-VPR, and HF-VPR system) by using short 15-bp sgRNA for gene activation and long 23-bp sgRNA for gene editing (Fig. 4d). To this end, we designed two experiments, one for *MYOD* activation and *IL1RN* editing, the other for *IL1RN* activation and *MYOD* editing. Deep-seq assays showed that all the three *As*Cas12a nucleases cleaved genomic DNA at the *MYOD* promoter region with comparable levels when using a 23-bp sgRNA, and failed to induce indels when using a 15-bp sgRNA. For activation, we observed that *MYOD* expression can be activated to a similar extent with the WT-, RKA-, and HF-VPR systems when transfected with 15-bp sgRNAs (Fig. 4e). And similar results were observed at the *IL1RN* site (Fig. 4e). However, we also noticed that 23-bp guides could activate transcription, although to a less extent than 15-bp guides (Fig. 4e), which was consistent with a previous report [71]. Cas12a usually cleaves DNA at around 18–23 bps distant from its PAM [29, 72], and DNA repair after cleavage could form a new imperfect matched sgRNA targeting site with mismatches at the sgRNA distal end. Since 15-bp sgRNA is able to activate gene expression, distal-mismatched 23-bp sgRNA might guide Cas12a to the repaired site using the proximal matched sequence and activate gene expression. Together, these data indicated that the RKA-VPR system could mediate gene activation and editing with different lengthed sgRNAs.

### AsCas12a-RKA holds editing safety in disruption oncogenic BRAF^V600E^

**Fig. 5:**
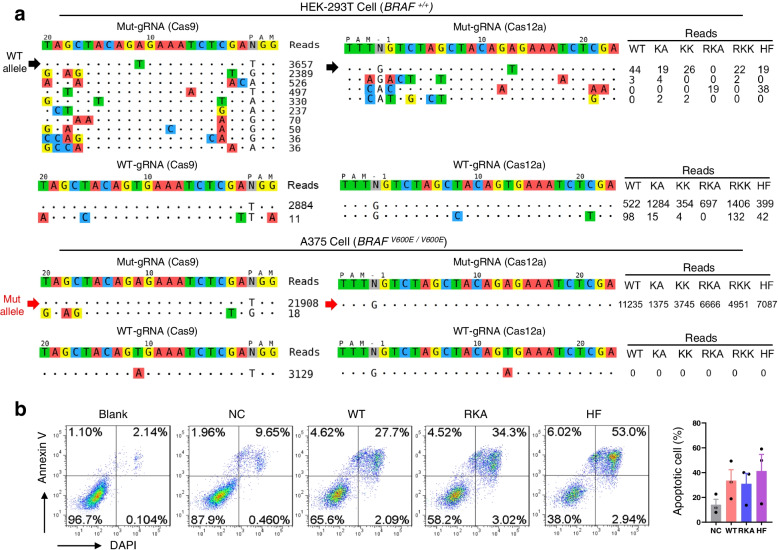
Disruption of the oncogenic BRAF^V600E^ mutation with *As*Cas12a variants in human A375 melanoma cells. **a** Tag-seq assessing the editing specificity of *As*Cas12a-WT and *As*Cas12a variants for BRAF with WT- and mut-sgRNAs in HEK-293T Cell (*BRAF* ^*+/+*^) and A375 Cell (*BRAF* ^*V600E/V600E*^). **b** Flow cytometry detecting the cells apoptosis induced by disrupting the BRAF^V600E^ mutation using *As*Cas12a-WT, *As*Cas12a-RKA, and *As*Cas12a-HF. Blank, A375 cells without treatment. NC, *As*Cas12a-WT, *As*Cas12a-RKA, and *As*Cas12a-HF mix + sgRNA-EGFP. WT/RKA/HF, *As*Cas12a-WT/*As*Cas12a-RKA/*As*Cas12a-HF + mut-BRAF-sgRNA. Mean values are presented with SEM, *n*=3 independent experiments

BRAF-V600E (1799T>A) is one of the most frequently reported driver mutations in multiple types of cancers, and patients with such mutations could benefit from disrupting this mutant allele [39, 73]. However, a major concern for implementing CRISPR/Cas9 for gene therapy is the relatively high frequency of off-target effects. Therefore, we sought to examine the therapeutic potential of the high-fidelity Cas12a-RKA for editing this mutation. Melanoma cell line A375 is a homozygous genotype with BRAF-V600E [74] (Additional file 2: Figure S5a). By using a mut-sgRNA, Tag-PCR assay [65] roughly displayed that the *As*Cas12a nucleases (WT, RKA, and HF) retained high editing selectivity at this site, as they did not cut wild-type but mutated sequence, whereas Cas9 recognized and cut both wild-type and mutated alleles (Additional file 2: Figure S5b, c). Next, to more accurately assess the editing selectivity of the Cas-nucleases (*Sp*Cas9-WT, *As*Cas12a-WT, and all the *As*Cas12a mutants in this study), Tag-seq experiments were performed by using the Cas9- and Cas12a-sgRNA (both contained WT- and mut-sgRNAs) in both *BRAF*^*+/+*^ HEK293T and *BRAF*^*V600E/V600E*^ A375 cells (Fig. 5a). Consistent with the Tag-PCR results, *Sp*Cas9 edited both wild-type and mutant BRAF with the mut-sgRNA and induced abundant off-targets editing (Fig. 5a), indicating a low selectivity for this mutation editing. In contrast, wild-type *As*Cas12a and the engineered variants displayed higher specificity with only few cleavages in wild-type BRAF and no off-targets detection in mutant BRAF when applied with the mut-sgRNA (Fig. 5a). However, among all the tested *As*Cas12a nucleases, variant RKA showed high-specificity and high-activity at this site (Fig. 5a). Further, by disruption *BRAF*^*V600E*^, *As*Cas12a-RKA induced A375 cell apoptosis with comparable level to *As*Cas12a-WT and *As*Cas12a-HF (Fig. 5b), demonstrating the therapeutic potential of *As*Cas12a-RKA to treat *BRAF*^*V600E*^ tumors.

### Engineering of high-performance LbCas12a variants via analogous substitutions to AsCas12a-RKA

**Fig. 6:**
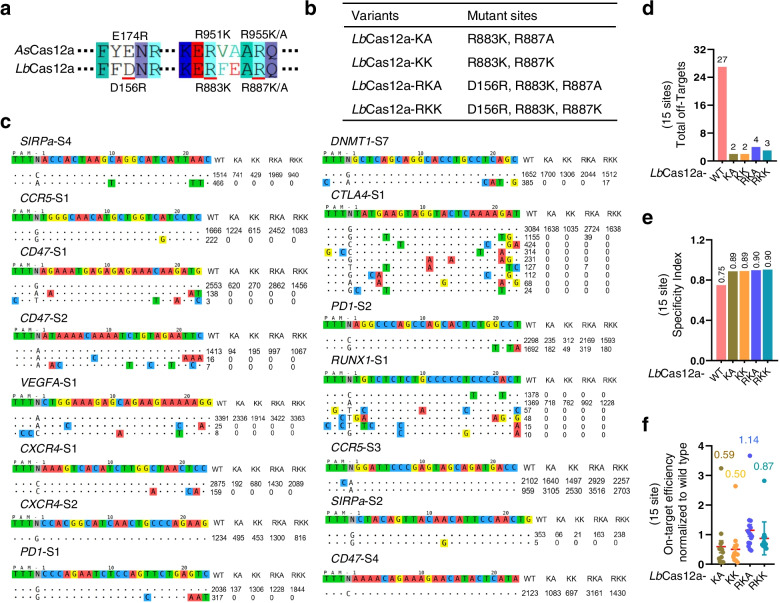
Generation of high-performance *Lb*Cas12a variants.** a** The conserved sites in *As*Cas12a and *Lb*Cas12a, underlined with red. **b** The mutation sites of *Lb*Cas12a variants. **c** Tag-seq-based comparative analyses of wild-type *Lb*Cas12a (WT), *Lb*Cas12a variant KA (KA), *Lb*Cas12a variant KK (KK), *Lb*Cas12a variant RKA (RKA), and *Lb*Cas12a variant RKK (RKK) with fifteen sgRNAs targeting nine genes. **d** Total number of off-target sites detected with the fifteen sgRNAs. **e** Specificity index (value was calculated by the ratio of total on-target reads to the on-target reads plus the off-target reads within the fifteen sites). **f** Normalization of on-target activity of *Lb*Cas12a-KA, *Lb*Cas12a-KK, *Lb*Cas12a-RKA, and *Lb*Cas12a-RKK to wild-type *Lb*Cas12a

Encouraged by *As*Cas12a-RKA, we next examined whether these analogous positions in *As*Cas12a-RKA could be deployed in *Lb*Cas12a, another commonly used Cas12a nuclease, to generate *Lb*Cas12a mutants with high performance as well. Via amino acid sequence alignment between *As*Cas12a and *Lb*Cas12a, we identified the conserved amino acid residues and constructed four *Lb*Cas12a variants, KK, KA, RKA, and RKK (Fig. 6a, b). Western blotting showed similar protein expression levels of these variants (Additional file 2: Figure S6). Then, we compared the activity and specificity of the four *LbCas12a* variants to WT *Lb*Cas12a by Tag-seq with fifteen sgRNAs targeting nine human endogenous genes (Fig. 6c). Consistently, the total off-targets of *Lb*Cas12a-KK, *Lb*Cas12a-KA, *Lb*Cas12a-RKA, and *Lb*Cas12a-RKK reduced from 27 to 2, 2, 4, and 3, respectively (Fig. 6d). The specificity of these four mutants was increased as well (Fig. 6e). Notably, similar to *As*Cas12a-RKA, *Lb*Cas12a-RKA showed high efficiency and specificity (Fig. 6c–f).

Collectively, all the above results demonstrated that the engineered Cas12a-RKA variant behaved as a high-active and high-specific nuclease; we hence termed it as “Cas12a-Plus”.

### Systematical comparison of available high-active AsCas12a variants

**Fig. 7:**
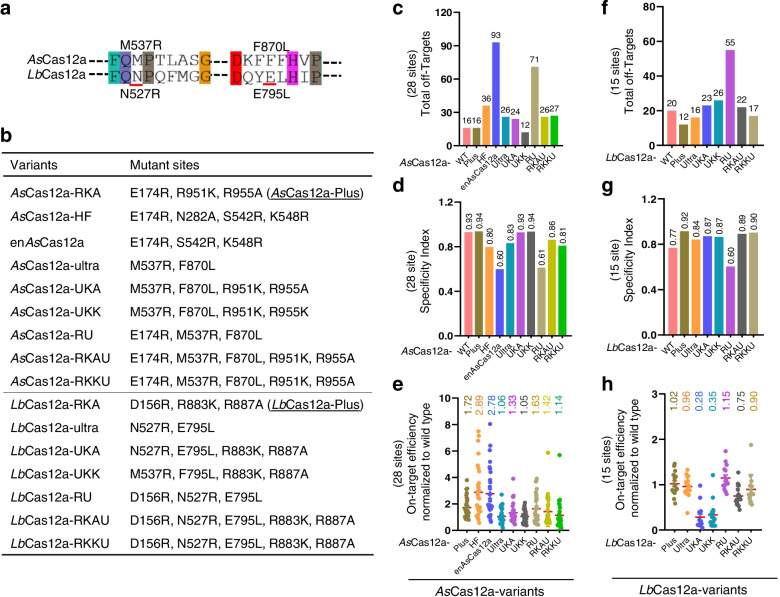
Performance comparison of Cas12a variants. **a** The conserved sites of ultra variant in *As*Cas12a and *Lb*Cas12a, underlined with red. **b** The mutation sites of *As*Cas12a and *Lb*Cas12a variants. RKA=Plus, UKA=ultra+KA, UKK=ultra+KK, RU=high-active substitution E174R/D156R+ultra, RKAU=RKA+ultra, RKKU=RKK+ultra. **c–e** Tag-seq-based comparative analyses of wild-type *As*Cas12a (WT), and *As*Cas12a variants with twenty-eight sgRNAs targeting nineteen genes (see Additional file 2: Figure S7b). **c** Total number of off-target sites detected with the twenty-eight sgRNAs. **d** Specificity index (value was calculated by the ratio of total on-target reads to the on-target reads plus the off-target reads within the twenty-eight sites). **e** Normalization of on-target activity of *As*Cas12a variants to wild-type *As*Cas12a. **f–h** Tag-seq-based comparative analyses of wild-type *Lb*Cas12a (WT), and *Lb*Cas12a variants with fifteen sgRNAs targeting nine genes (see Additional file 2: Figure S8b). **f** Total number of off-target sites detected with the fifteen sgRNAs. **g** Specificity Index (value was calculated by the ratio of total on-target reads to the on-target reads plus the off-target reads within the fifteen sites). **h** Normalization of on-target activity of *Lb*Cas12a variants to wild-type *Lb*Cas12a

When our manuscript was underwriting, a new *As*Cas12a variant, *As*Cas12a-ultra, was reported with significantly enhanced activity [52]. To systematically compare the performance among these high-active Cas12a variants, we constructed the *As*Cas12a-ultra, and the *Lb*Cas12a-ultra that was created by amino acid sequence conservation (Fig. 7a). Correspondingly, more variants were generated by combining with the high-fidelity mutant sites KK and KA, or by introducing the high-active substitutions, or by combining the RKA and RKK mutations (Fig. 7b). After confirming the comparable protein expression level by Western blotting (Additional file 2: Figures S7a and S8a), we comprehensively analyzed their performance using Tag-seq with twenty-eight sgRNAs targeting nineteen genes among *As*Cas12a variants (Additional file 2: Figure S7b), and with fifteen sgRNAs targeting nine genes among *Lb*Cas12a variants (Additional file 2: Figure S8b), respectively. As a result, although the mutant en*As*Cas12a and its high-fidelity version en*As*Cas12-HF exhibited the highest editing abilities, they induced numbers of extra off-targets, particularly the en*As*Cas12a (Fig. 7c–e). Surprisingly, the *As*Cas12a-ultra showed a slightly improved activity and an obvious decreased specificity (Fig. 7c–e), and disruption of mNeonGreen expression in HEK293T-KI reporter cell line leads to similar results (Additional file 2: Figure S9), which was different from the previous report [52]. We speculated that the possible reason was the difference in delivery approach. RNP delivery and plasmid transfection were employed by the previous report and the current study, respectively. And these two methods had been demonstrated to result in different efficiency of gene editing [75, 76]. When introduced the high-active mutation E174R into the *As*Cas12a-ultra (termed RU), efficiency was increased while specificity was significantly compromised (Fig. 7c–e). All the variants containing the KA and KK mutations exhibited improved specificity. Notably, among the ten tested variants of *As*Cas12a, *As*Cas12a-Plus displayed the best balance in editing performance with moderately enhanced activity and specificity (Fig. 7c–e). And similar results were observed in the *Lb*Cas12a versions (Fig. 7f–h, Additional file 2: Figures S8 and S10).

## Discussion

The off-target effect of the CRISPR-Cas genome editing tools is a major concern for therapeutic applications. It has been reported that Cas12a exhibits a higher specificity over the widely used *Sp*Cas9; however, the relatively low activity restricts their broad use [26, 27, 29, 30, 44]. Given the advantageous properties of the Cas12a nuclease, such as the higher specificity and distinct PAM preference [29, 31], it represents a powerful alternative for gene editing. Here, we constructed novel Cas12a variants termed “Cas12a-Plus” by rational structure-guided engineering to enable more potent and more specific gene editing.

Since Cas12a and Cas9 are complementary to each other as genome editing tools, like Cas9, Cas12a has also attracted plenty of effort for protein engineering to expand editing range, enhance activity, and improve specificity (for detailed information, please refer to Additional file 1: Table S1 and Table S2). For instance, *As*Cas12a-RVR and *As*Cas12a-RR have been engineered to expand the PAM recognition range and their high-fidelity derivative mutants *As*Cas12a-RVRA and *As*Cas12a-RRA can improve editing specificity but with compromised activity [26, 27, 41]. More recently, enhanced *As*Cas12a (en*As*Cas12a/en*As*Cas12a-HF) [27], *As*Cas12a-ultra [52], and the imp*Lb*Cas12a [45] have been reported to significantly enhance editing efficiency and broaden editing ranges. However, our data (Figs. 2, 3, and 7) and the previous reports [27, 45, 52] demonstrate that these variants induce extra off-targets cleavages, indicating a compromise of specificity. In this study, we created alternative high-fidelity Cas12a-KA and KK, and high-active and high-specific Cas12a-Plus (Figs. 1, 2, 3, and 7), which expanded the Cas12a toolbox. Therefore, we recommend to use Cas12a variants with different properties according to the intended applications. Our study provides Cas12a-Plus as the first variant with enhanced activity and increased specificity, which holds great potential for broad applications, especially for clinical disease therapy.

With unique features, Cas12a outperforms Cas9 in some applications. For example, Cas12a has been reported to be better than Cas9 for one-step generation of modular CAR-T cells [77]. With the crRNA self-processing activity, Cas12a could be used for combinatorial genetic screening [50, 51]. In this study, we also found that, with higher specificity, Cas12a outperformed Cas9 to disrupt *BRAF*^*V600E*^ mutated allele and thus triggered cancer cell death with much less genome editing in normal cells (Fig. 5). With enhanced specificity and activity, the *As*Cas12a-Plus variant further improved the performance (Fig. 5). Since it maintained the enhanced-activity and improved-specificity in multiplex editing and transcriptional activation (Figs. 3 and 4), we believe that *As*Cas12a-Plus could outperform wild-type *As*Cas12a in combinatorial genetic screening as well as other Cas12a-based applications.

According to the energy hypothesis [22, 23], the Watson-Crick base pairing between gRNA and the target DNA strand as well as the binding between Cas protein and the PAM bases provided specific energy (A, T, C, and G base-dependent), while the binding between Cas protein and the backbone of target DNA strand and PAM DNA as well as the binding between Cas protein and non-target DNA strand provided non-specific energy (base-independent). Both specific and non-specific energy contributed to recognition and cleavage. Besides, it has been reported that Bridge helix arginines (Rs) play a critical role in sensitivity to mismatched sequences [69]. Based on these, we finally focused on R951 and R955 by analyzing the crystal structure of the *As*Cas12a-crRNA-target DNA complex [64]. Because they were located at Bridge helix and seemed to provide non-specific contacts [64]. Since R951A mutation reduces Cas12a activity [64], we thus used R951K as an alternative, since lysine (K) is highly similar to arginine (R) in structure and is of less possibility to form hydrogen bonds between Cas12a and the target DNA strand as predicted by the complex structure. We did demonstrate that R951K/R955A (KA) and R951K/R955K (KK) mutation could improve editing specificity (Fig. 1). By combining with a known activity-enhancing E174R mutation [27], we created *As*Cas12a-Plus with high-activity and high-fidelity, which was also applicable with *Lb*Cas12a (Figs. 2 and 6). Other variants combined with the RKA mutation, such as the *As*Cas12a-ultra, could improve activity and specificity (the RU and RKAU variants, Fig. 7). In theory, this strategy could be introduced into other Cas12a variants, such as the PAM-less-restricted RVR and RR mutations [26, 27, 41], or combined with the chemical modification [10, 15], to improve performance. Amino acid residues other than the tested E174/K780/R951/R955 could contribute to the activity and specificity of *As*Cas12a, such as the residues contacting the non-target DNA strand or the crRNA, and thus mutating these residues might improve *As*Cas12a performance. All of these hypotheses need to be tested in the future.

## Conclusions

In summary, we created novel *As*Cas12a and *Lb*Cas12a variants with both high-activity and high-fidelity, expanding the Cas12a toolbox, and thus, these variants could enhance the performance of Cas12a in a wide spectrum of applications.

## Methods

### Plasmid construction

*As*Cas12a and *Lb*Cas12a variants expression plasmids bearing amino acid substitutions were generated by standard PCR and molecular cloning into a plasmid contained a CAG promoter, HA, P2A-mcherry cassette via Gibson Assembly. sgRNA expression plasmids were constructed by ligating oligonucleotide duplexes into EcoR V and Hind III cut pBlueScript backbone with a human U6 promoter and an *As*- or *Lb*-crRNA sequence. All the plasmids were confirmed by Sanger sequencing, and all the sgRNAs used in this study are shown in Additional file 1: Table S3.

### Cell culture and cell transfection

HEK293T, B16, and A375 cells were maintained in Dulbecco’s modified Eagle’s medium (DMEM, Life Technologies) at 37°C in a 5% CO2 humidified incubator. All growth media were supplemented with 2 mM L-glutamine (Life Technologies), 100 U/mL penicillin, 100 μg/mL streptomycin (Life Technologies), and 10% fetal bovine serum. All the cell lines in this study were cultured no more than 10 passages.

Cells were transfected with PEI reagent (Polysciences, Inc., PA, USA) according to the manufacturer’s instructions. Briefly, 250 ng of pCAG-Cas12a-mcherry and 250 ng of sgRNA-encoding plasmids were transfected per well in a 24-well plate. Cells were harvested 2–3 days after transfection, then the genomic DNA or the total RNA were extracted for the following assays.

### Tag-seq method

Tag-seq experiments were performed and analyzed as previously described [65]. Briefly, HEK293T cells were transfected by PEI with 20 nM Tag, 1000 ng of Cas nuclease, and 1000 ng single sgRNA or a pool sgRNAs (30–50 ng/sgRNA) per well in a six-well plate. A375 cells were transfected by Amaxa Cell Line Nucleofector Kit V (VCA-1003, LONZA, Switzerland) following the manufacturer’s instructions (2D) with 20 nM Tag, 1200 ng of Cas nuclease, and 800 ng WT/Mut-BRAF-sgRNA. All cells were harvested 3 days after transfection and genomic DNA was extracted for one-step libraries preparation by the Fragmentation, End Preparation, and dA-Tailing Module and Adapter Ligation Module kit (Vazyme Biotech Co., Ltd., Nanjing, China). The R and L libraries were constructed by PCR with library preparation primers, which were followed by sequencing (Hiseq/NovaSeq platform, Novogene, Beijing, China) and analysis with a Tag-seq bioinformatics pipeline. Tag-seq experiments were performed with the same input gDNA and an equal sequencing depth. The analysis pipeline is available at https://github.com/zhoujj2013/Tag-seq and 10.5281/zenodo.4679460.

### Activity and specificity scoring

For the comparisons of performance among Cas12a variants, Tag-seq reads were used for calculating the editing activity and targeting specificity. Activity scores were calculated as the mean ratio of the on target reads across all the tested sites, normalized to the WT Cas12a nuclease. Specificity scores were calculated as the ratio of the on target reads to the on-target reads plus the off-target reads across all the tested sites.

### Tag-PCR assay

Tag-PCR was used to roughly determine the editing events of the CRISPR-Cas systems, which reflected the insertion efficiency of the Tag at the editing sites [65]. Briefly, cell transfection was the same as the Tag-seq method. After extraction of genomic DNA with integrated Tag sequence at break sites, PCR was performed by using the Tag-specific primer and a locus-specific R primer, then PCR products were assessed by running on an agarose gel.

### Deep-seq analysis

Deep-seq was used to determine the indel frequency. Briefly, the primers with forward and reverse indexes were used to amplify the genomic regions in the first-round PCR. Then, equal amounts of the first PCR products were mixed and subjected to a second round of PCR with the P5- and P7-containing primers to generate the sequencing libraries. Paired-end sequencing was performed using the Hiseq/NovaSeq system (Novogene, Beijing, China). Indel frequency was calculated as the ratio of (read counts with indel sequence)/(total sequencing read counts). The deep-seq primers and the samples’ index information were listed in Additional file 1: Tables S4 and S5.

### T7EI and polyacrylamide gel electrophoresis (PAGE) assay

For T7EI analysis, the amplicons were purified, denatured at 95°C for 5 min and annealed in NEB Buffer 2 with a slow ramp down (approximately −2°C/min) to 4°C, then subjected to T7 endonuclease I (NEB, UK) digestion for 3 h at 37°C before loading on a 2% agarose gel. For PAGE assay, genomic DNA was isolated using sarkosyl lysis buffer (10 mM Tris pH7.6, 0.5% Sarkosyl, 10 mM NaCl, 10 mM EDTA, 0.1 mg/ml proteinase K) and the target sites were amplified by PCR. The purified amplicons were reannealed to form heteroduplexes and then subjected to 5% polyacrylamide gel electrophoresis. All T7EI and the PAGE primers in this study are listed in Additional file 1: Table S4.

### Quantitative real-time PCR

Total RNA from the transfected cells was isolated using Trizol Reagent (Thermo Fisher, USA) following the manufacturer’s instructions. Total RNA (1 μg) was reverse transcribed into cDNA and then quantitative real-time PCR (SYBR Premix Ex Taq II, TAKARA, China) was performed using a LightCycler 96 System (Roche, Switzerland). Relative gene expression was calculated using the 2^−ΔΔCt^ method after normalizing to GAPDH expression. All the qPCR primers are listed in Additional file 1: Table S4.

### Western blotting

To detect the expression of the *As*Cas12a and *Lb*Cas12a variants, the transfected cells were lysed in a 2×SDS loading buffer and boiled for 10 min. Lysates were resolved through SDS/PAGE and transferred onto a nitrocellulose membrane which was blocked using 5% non-fat milk and sequentially incubated with primary antibodies (anti-HA, sigma, USA, anti-GADPH, Proteintech, China) and an HRP-conjugated horse anti-mouse IgG secondary antibody (CST, USA, CAT# 7076S). All the probed proteins were finally detected through chemiluminescence following the manufacturer’s instructions (Pierce, USA).

### RNA-seq

RNA-seq experiments were performed and analyzed as previously described [78]. Briefly, total RNA was extracted by Trizol reagent (Invitrogen, Carlsbad, CA, USA), then mRNA was used for the standard RNA libraries’ preparation, and libraries were sequenced by 150 bp paired-end Novaseq device. For data analysis, Hisat2 v2.0.52 was used to build the index of the reference genome and align the paired-end clean reads with the reference genome. Then, StringTie v2.23 was used to count the read numbers mapped to each gene. Fragments per kilobase per million (FPKM) of each gene was calculated based on the length of the gene and the reads count mapped to this gene. Differential expression was defined by a Benjamini-Hochberg adjusted *p*-value (*q* value | FDR) of <0.05 and a fold change of >2 or <0.5. All figures were plotted using R package ggplot2.

### FACS analysis

All flow cytometry analyses were performed using FlowJo software (TreeStar, USA). Cells were harvested 48 h post-transfection, and the cleavage efficiency of *As*Cas12a variants was determined as the proportion of GFP negative cells within the *As*Cas12a-transfected cells (mCherry-positive). To detect the apoptosis of A375, cells were first transduced with the lentivirus encoding the *As*Cas12a-WT, *As*Cas12a-RKA, and *As*Cas12a-HF protein, then cells were co-transfected with the mut-BRAF or EGFP (as a negative control) sgRNA and a pCMV-mCherry reporter plasmid. After 7 days, cells were gated out using mCherry, followed by the standard procedures of the Annexin V-FITC Apoptosis Detection Kit (BestBio, China) according to the manufacturer’s instructions.

### Statistics analysis and reproducibility

Student’s *t*-test and one-way ANOVA were used in this study for the statistical analysis. The reproducibility was shown by performing two-four independent biological replicate experiments.

## Supplementary Information

**Additional file 1. **Supplementary Information, including supplementary tables and DNA and amino acid sequences. **Table S1.** The engineering of the CRISPR-*As*Cas12a/*Lb*Cas12a systems. **Table S2.** The engineering of the CRISPR-Cas12a (excluded *As*Cas12a and *Lb*Cas12a) systems. **Table S3.** The sgRNAs used in this study. **Table S4.** Primers for this study (including qPCR, T7E1/PAGE, Deep-seq). **Table S5.** Index information for deep-seq. **Supplementary sequences.** DNA and Amino acid sequences used in this study.

**Additional file 2: Figure S1.** Construction of *As*Cas12a variants and assessment of their specificity. **Figure S2.** Specificity assessment of *As*Cas12a-RKA. **Figure S3.** Specificity comparison of *As*Cas12a-WT, -RKA, -RKK, and -HF by Tag-seq. **Figure S4.** The Engineering of crRNA to improve efficiency. **Figure S5.** Editing of BRAF^V600E^ with *Sp*Cas9 and *As*Cas12a nucleases. **Figure S6.** Detection the expression levels of *Lb*Cas12a nucleases. **Figure S7.** Specificity assessment of *As*Cas12a-WT, -Plus, -HF, en*As*Cas12a, -ultra, -UKA, -UKK, -RU, -RKAU, and -RKKU by Tag-seq. **Figure S8.** Specificity assessment of *Lb*Cas12a-WT, -Plus, -ultra, -UKA, -UKK, -RU, -RKAU, -RKKU by Tag-seq. **Figure S9.** Editing efficiency analyses of *As*Cas12a-WT, -Plus, -HF, en*As*Cas12a, -ultra, -UKA, -UKK, and -RU. **Figure S10.** Editing efficiency analyses of *Lb*Cas12a-WT, -Plus, -ultra, -UKA, -UKK, -RU, -RKAU, and -RKKU.

**Additional file 3.** The supporting data values for the figures, including Figs. 1e, f, 2c, e, f, 3d, e, 4b, e, 5b, 6e, f, 7d, e, g, h, and Figs. S1c, S4b, c, S9, S10.

## Data Availability

All data generated or analyzed during this study are included in this published article, the sequencing data are deposited at NCBI SRA (Bioproject PRJNA755186), and the supporting data values for the figures are provided at Additional file 3.

## Abbreviations

CRISPR: Clustered regularly interspaced short palindromic repeats
Cas: CRISPR associated protein
HF: High-fidelity
ssDNA: Single-stranded DNA
T7E1: T7 endonuclease I
PAGE: Polyacrylamide gel electrophoresis
WT: Wild-type
en*As*Cas12a: Enhanced *As*Cas12a
dCas12a: DNase-inactive Cas12a
PAM: Protospacer-adjacent motif
