# Supplementary material for: Engineered Cas12a-Plus nuclease enables gene editing with enhanced activity and specificity
Source: BMC Biol. 2022 Apr 25;20:91. doi: 10.1186/s12915-022-01296-1 (PMC9040236; doi:10.1186/s12915-022-01296-1)
Supplement: Supplementary file 1 — Additional file 1. Supplementary Information, including supplementary tables and DNA and amino acid sequences. Table S1. The engineering of the CRISPR-AsCas12a/LbCas12a systems. Table S2. The engineering of the CRISPR-Cas12a (excluded AsCas12a and LbCas12a) systems. Table S3. The sgRNAs used in this study. Table S4. Primers for this study (including qPCR, T7E1/PAGE, Deep-seq). Table S5. Index information for deep-seq. Supplementary sequences. DNA and Amino acid sequences used in this study. [file 12915_2022_1296_MOESM1_ESM.pdf]

Supplementary Figures

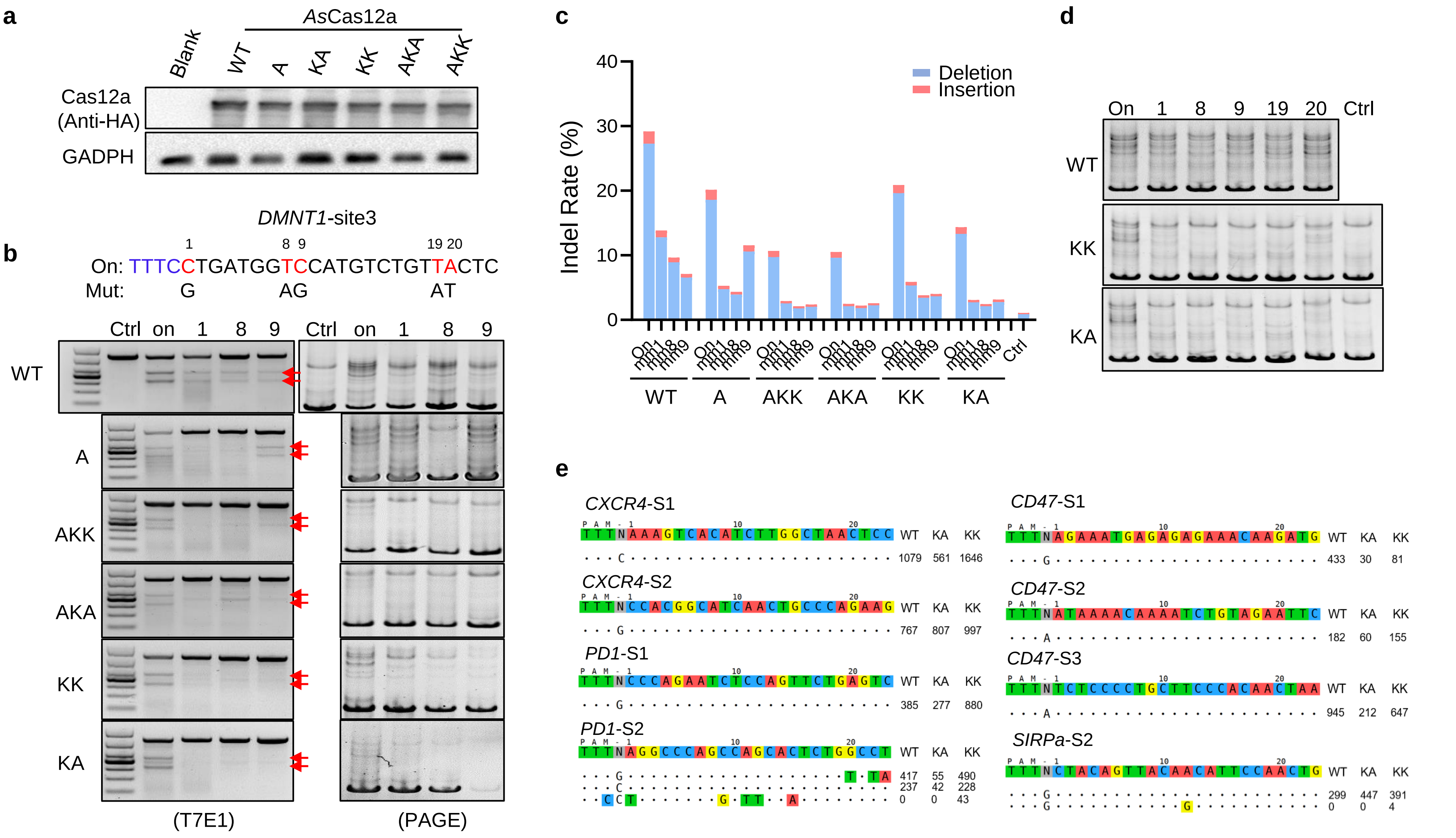

Figure S1. Construction of *As*Cas12a variants and assessment of their specificity

Figure S1. Construction of *As*Cas12a variants and assessment of their specificity. a Western blot showing the expression levels of *As*Cas12a-WT and *As*Cas12a variants. Blank, HEK293T cells without transfection. **b** T7E1 and PAGE gel assays assessing the editing activity of the *As*Cas12a variants at *DNMT1*-site3 with perfectly matched sgRNA or mismatched sgRNAs. PAM: highlighted in blue. On: on-target sgRNA. 1, 8, 9: the mismatched sgRNA with mutation sites in sgRNA that highlighted in red. Red arrows, cleavage products. **c** Deep-seq showing the editing efficiency of the *As*Cas12a variants at *DNMT1*-site3 with perfectly matched sgRNA or mismatched sgRNAs. **d** PAGE gel assay showing the editing activity of the *As*Cas12a-KK and *As*Cas12a-KA variants targeting *DNMT1*-site3 with sgRNAs with mismatched locations at 1, 8, 9, 19, and 20. **e** Tag-seq-based comparative analyses of wild-type *As*Cas12a (WT), *As*Cas12a variant KA (KA), and *As*Cas12a variant KK (KK) with seventeen sgRNAs targeting nine genes (also see Fig. 1c). HEK293T cells were co-transfected with the plasmids expressing *As*Cas12a variants and a pooled seventeen sgRNAs targeting nine genes. Genomic DNA was harvested after three days post-transfection for libraries construction and Tag-seq analysis. Read counts represented a measure of cleavage frequency at a given site, mismatched positions within the spacer or PAM are highlighted in color.

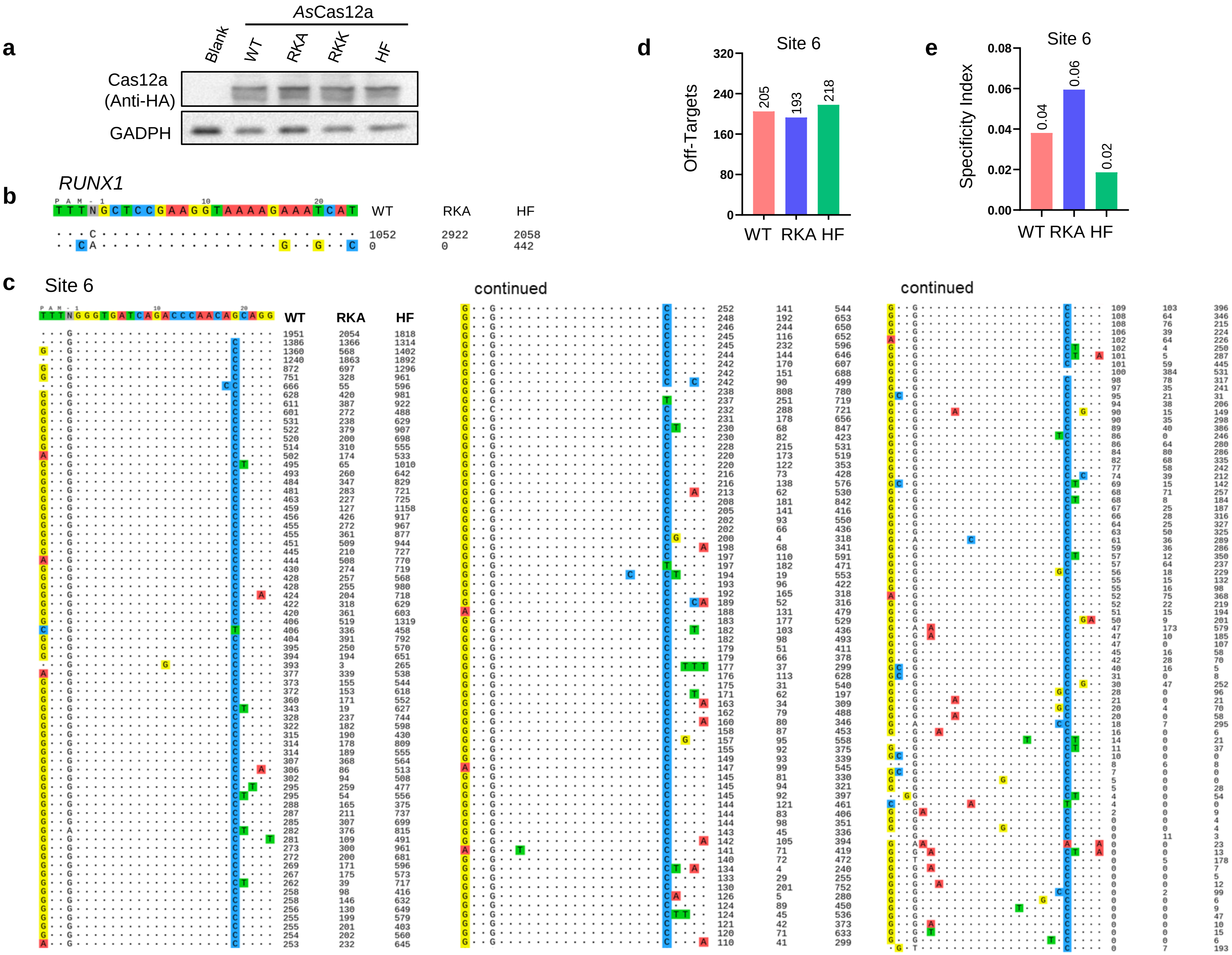

Figure S2. Specificity assessment of *As*Cas12a-RKA

Figure S2. Specificity assessment of *As*Cas12a-RKA. a Western blot showing the expression levels of *As*Cas12a-WT and high active *As*Cas12a variants. **b** On-target and off-target cleavage sites of wild-type *As*Cas12a (WT), *As*Cas12a variant RKA (RKA), and en*As*Cas12a-HF (HF, the high-specificity version of the en*As*Cas12a) targeting *RUNX1* as determined by Tag-seq. **c** On-target and off-target cleavage sites of WT, RKA, and HF targeting Site 6 as determined by Tag-seq. **d** Total number of off-target sites detected at Site 6. **e** Specificity Index (value was calculated by the ratio of total on-target reads to the on-target reads plus the off-target reads).

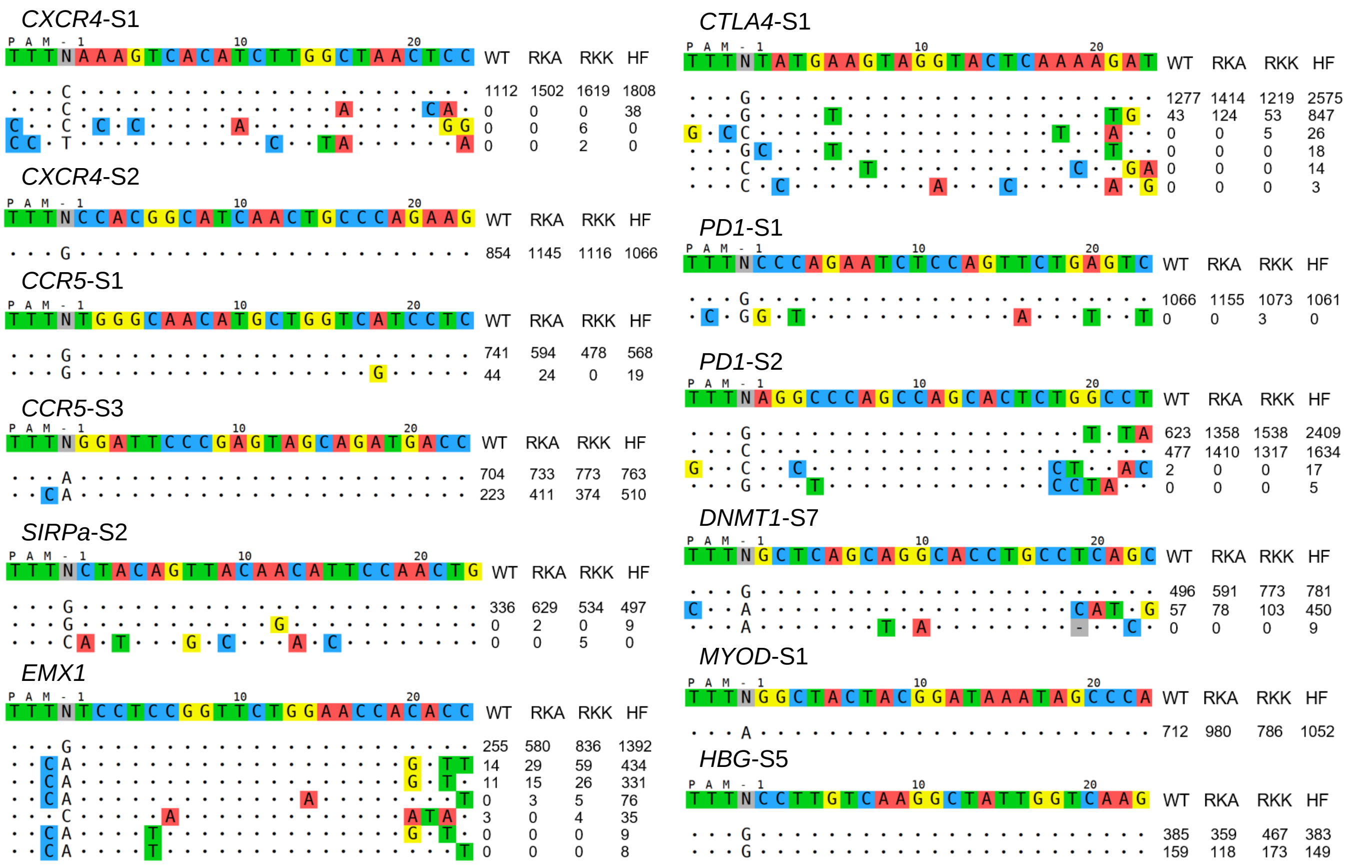

Figure S3. Specificity comparison of *As*Cas12a-WT, -RKA, -RKK, and -HF by Tag-seq

Figure S3. Specificity comparison of *As*Cas12a-WT, -RKA, -RKK, and -HF by Tag-seq. HEK293T cells were co-transfected with the plasmids expressing *As*Cas12a variants and a pooled twenty-two sgRNAs targeting twelve genes. Genomic DNA was harvested after three days post-transfection for libraries construction and Tag-seq analysis. Read counts represented a measure of cleavage frequency at a given site, mismatched positions within the spacer or PAM are highlighted in color. (also see Fig. 2b)

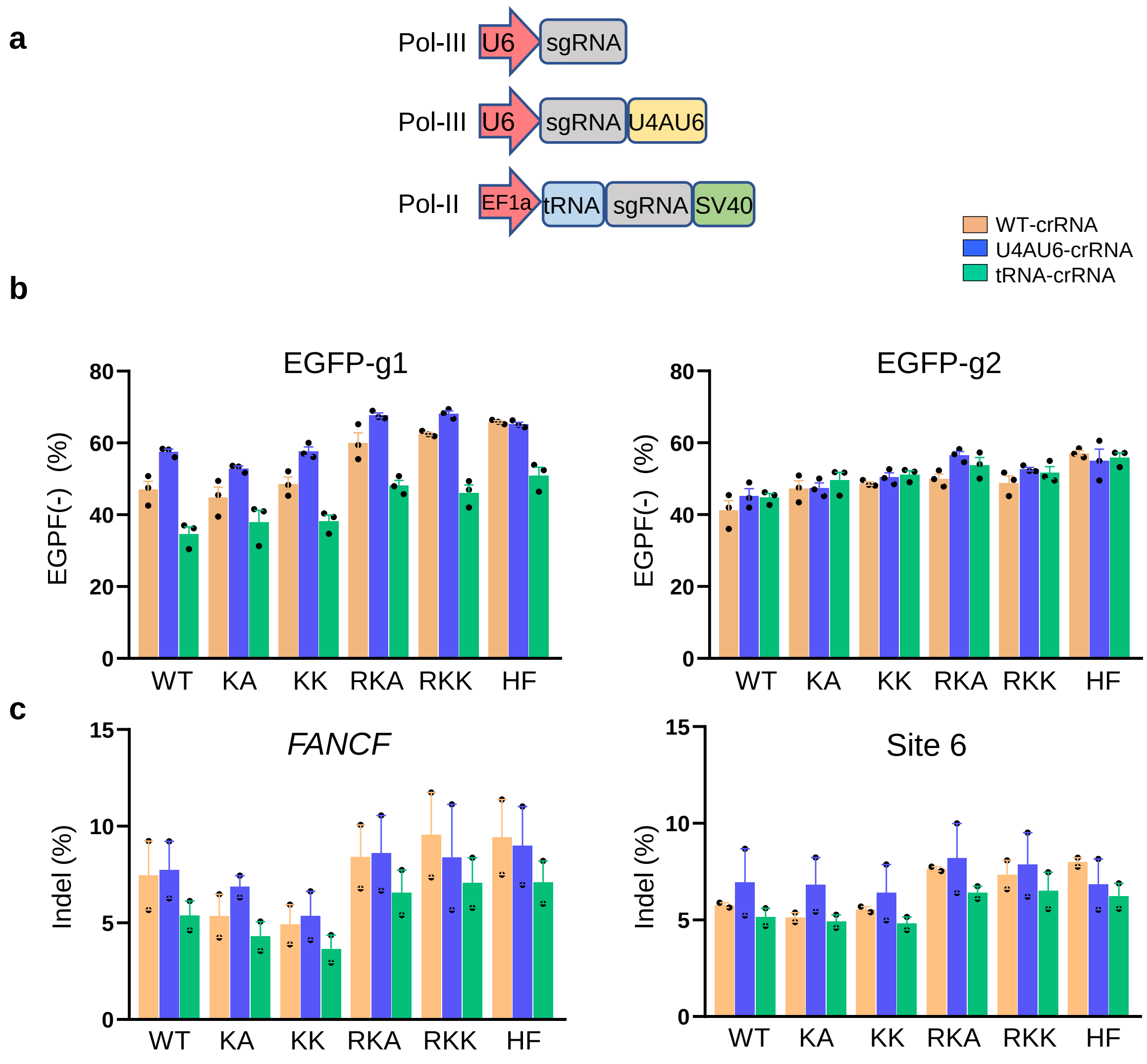

Figure S4. The Engineering of crRNA to improve efficiency

Figure S4. The engineering of crRNA to improve efficiency. a Schematic of sgRNA engineering. **b** Flow cytometry detected the efficiency of disruption EGFP by *As*Cas12a-WT and *As*Cas12a variant using the engineered sgRNAs. EGFP-g1/g2, sgRNA1/sgRNA2 targeting *EGFP*. Mean values are presented with SEM, n=3 independent experiments. **c** Deep-seq showed the editing efficiency of *As*Cas12a-WT and *As*Cas12a variants targeting human endogenous *FANCF* genes and Site 6 using engineered sgRNAs. n=2 independent experiments. Indel was revealed by Deep-seq.

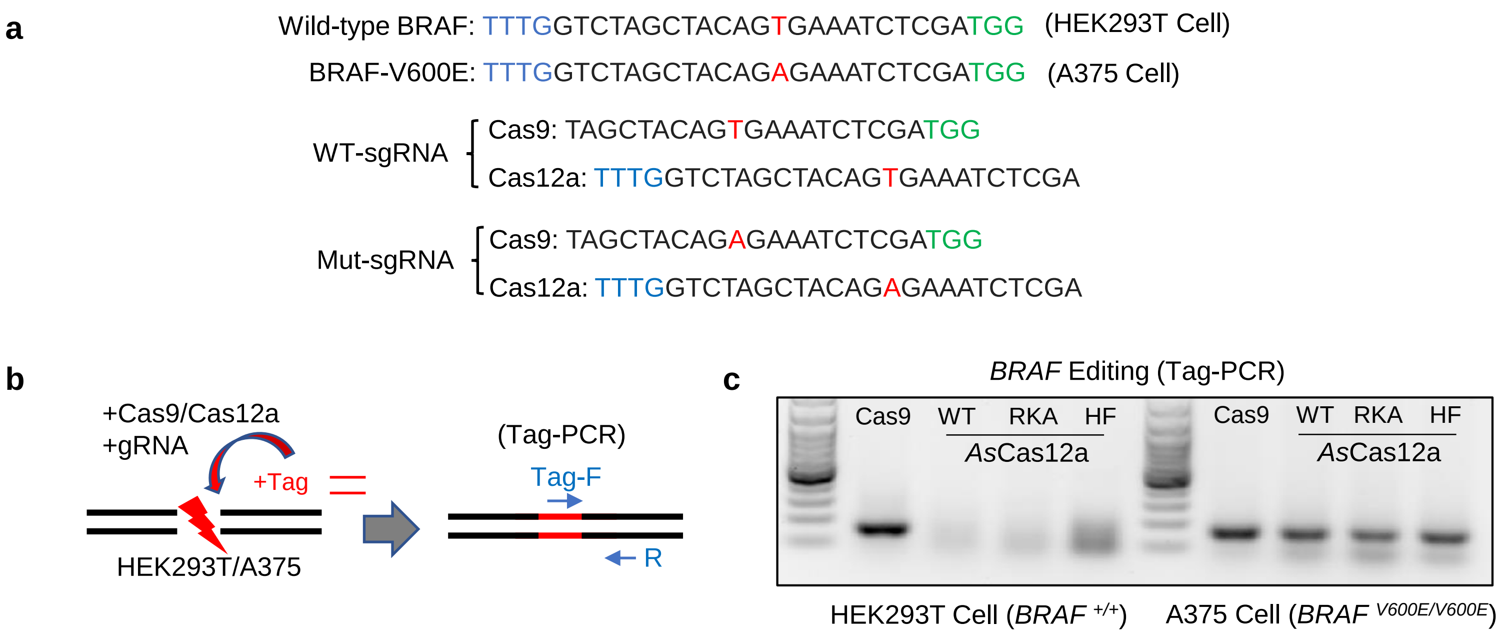

Figure S5. Editing of $BRAF^{V600E}$ with *Sp*Cas9 and *As*Cas12a nucleases

Figure S5. Editing of BRAF^{V600E} with *Sp*Cas9 and Cas12a nucleases. a Illustration of the *Sp*Cas9/Cas12a sgRNAs targeting the wild-type or mutant *BRAF. Sp*Cas9 PAM: highlighted in green. Cas12a PAM: highlighted in blue. Mutated site: highlighted in red. **b** Schematic of the Tag-PCR. Modified oligonucleotide DNA (Termed Tag) can be integrated into genomic DNA by Cas9/Cas12a nucleases. The editing efficiency can be roughly determined by PCR using the Tag specific primer and a genomic site-specific primer indicated with the blue arrows. **c** Tag-PCR showed the editing efficiency for the wild-type and mutant *BRAF* in HEK293T and A375 cell line with Cas9/Cas12a mut-sgRNA.

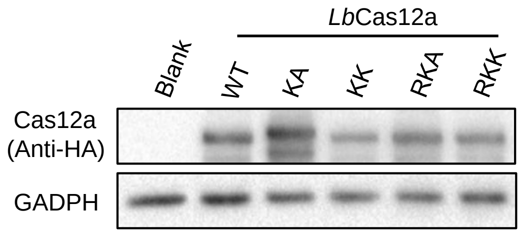

Figure S6. Detection the expression levels of *Lb*Cas12a nucleases

Figure S6. Detection the expression levels of *Lb*Cas12a nucleases. Western blot showing the expression levels of *Lb*Cas12a-WT and *Lb*Cas12a-variants. Blank, HEK293T without transfection

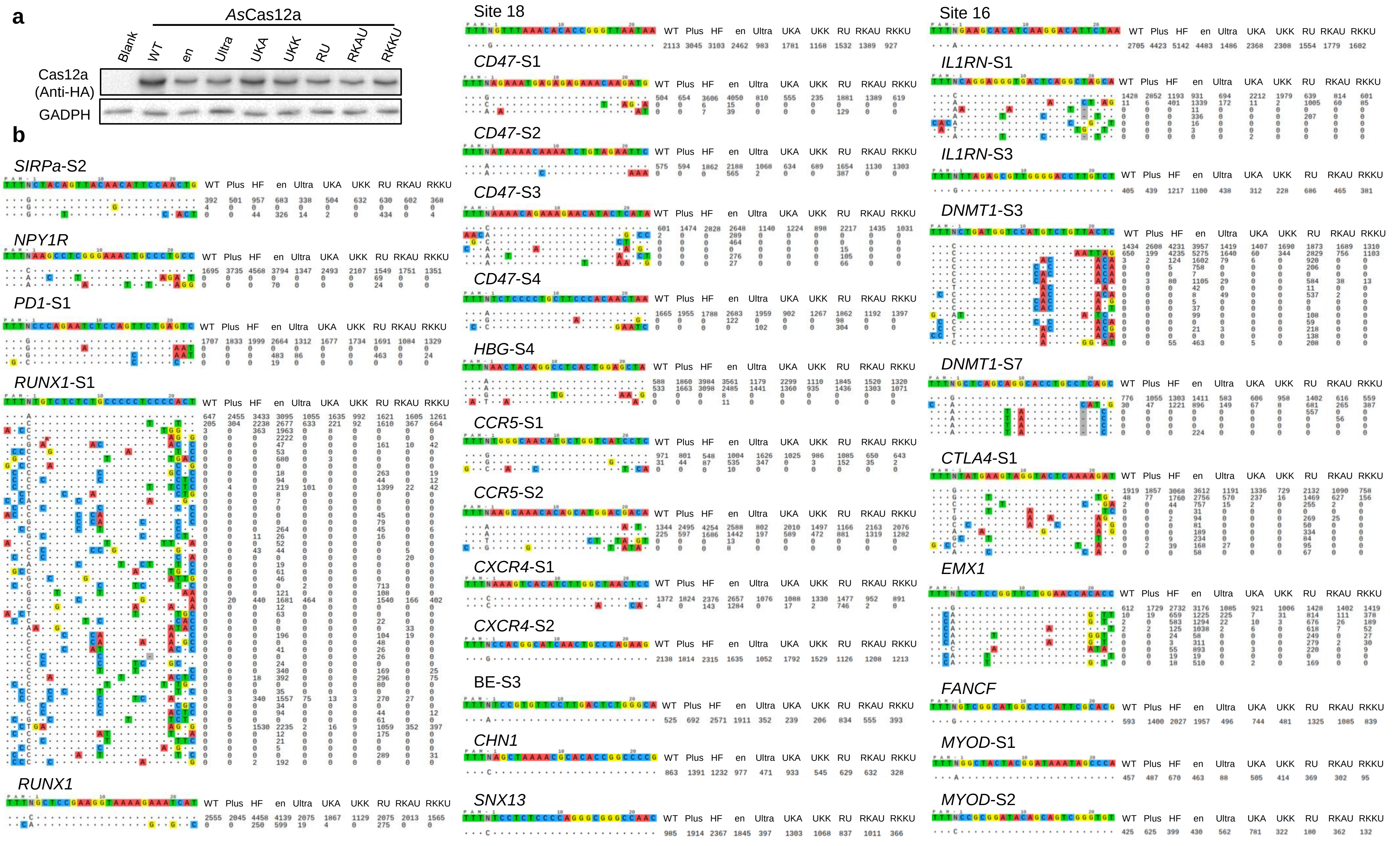

Figure S7. Specificity assessment of *As*Cas12a-WT, -Plus, -HF, en*As*Cas12a, -ultra, -UKA, -UKK, -RU, -RKAU, and -RKKU by Tag-seq

Figure S7. Specificity assessment of *As*Cas12a-WT, -Plus, -HF, en*As*Cas12a, -ultra, -UKA, -UKK, -RU, -RKAU, and -RKKU by Tag-seq. **a** Western blot showing the expression levels of *As*Cas12a variants. Blank, HEK293T without transfection. **b** Specificity assessment of *As*Cas12a variants by Tag-seq. HEK293T cells were co-transfected with the plasmids expressing *As*Cas12a variants and a pooled twenty-eight sgRNAs targeting nineteen genes. Genomic DNA was harvested after three days post-transfection for libraries construction and Tag-seq analysis. Read counts represented a measure of cleavage frequency at a given site, mismatched positions within the spacer or PAM are highlighted in color. RKA=Plus, en=en*As*Cas12a, UKA=Ultra+KA, UKK=Ultra+KK, RU=high active mutation E174R+Ultra, RKAU=RKA+Ultra, RKKU=RKK+Ultra.

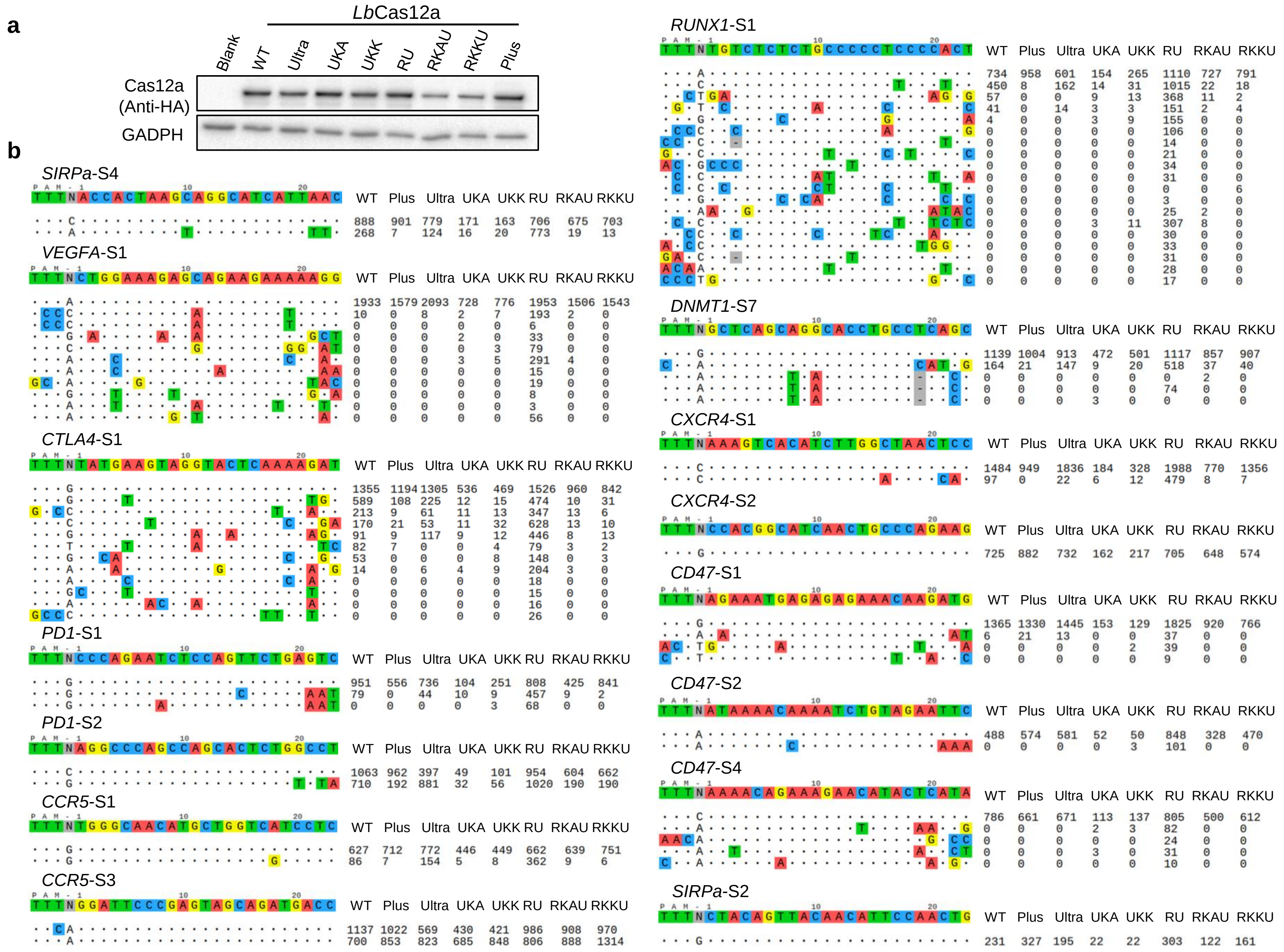

Figure S8. Specificity assessment of *Lb*Cas12a-WT, -Plus, -ultra, -UKA, -UKK, -RU, -RKAU, -RKKU by Tag-seq

Figure S8. Specificity assessment of *Lb*Cas12a-WT, -Plus, -ultra, -UKA, -UKK, -RU, -RKAU, and -RKKU by Tag-seq. **a** Western blot showing the expression levels of *Lb*Cas12a variants. Blank, HEK293T without transfection. **b** Specificity assessment of *Lb*Cas12a variants by Tag-seq. HEK293T cells were co-transfected with the plasmids expressing *Lb*Cas12a variants and a pooled fifteen sgRNAs targeting nine genes. Genomic DNA was harvested after three days post-transfection for libraries construction and Tag-seq analyses. Read counts represented a measure of cleavage frequency at a given site, mismatched positions within the spacer or PAM are highlighted in color. RKA=Plus, UKA=Ultra+KA, UKK=Ultra+KK, RU=high active mutation D156R+Ultra, RKAU=RKA+Ultra, RKKU=RKK+Ultra.

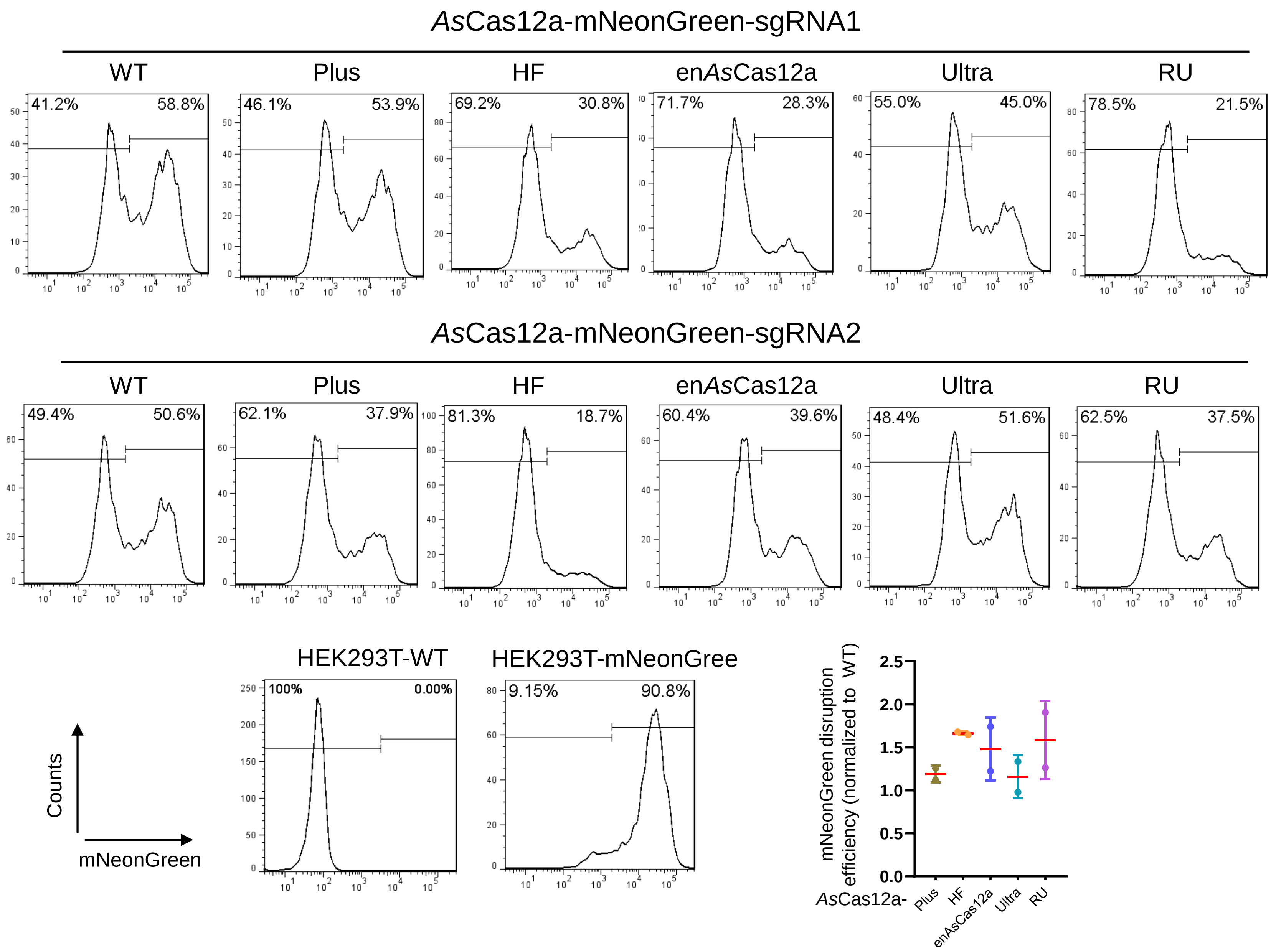

Figure S9. Editing efficiency analyses of *As*Cas12a-WT, -Plus, -HF, en*As*Cas12a, -ultra, -UKA, -UKK, and -RU

Figure S9. Editing efficiency analyses of *As*Cas12a-WT, -Plus, -HF, en*As*Cas12a, -ultra, -UKA, -UKK, and -RU. Flow cytometry detected the efficiency of disruption mNeonGreen by *As*Cas12a-WT and *As*Cas12a variants in HEK293T Knock-in mNeonGreen-PEST cell line. mNeonGree-sgRNA1/2, *As*Cas12a sgRNA targeting mNeonGreen site 1/2. n=2 independent experiments.

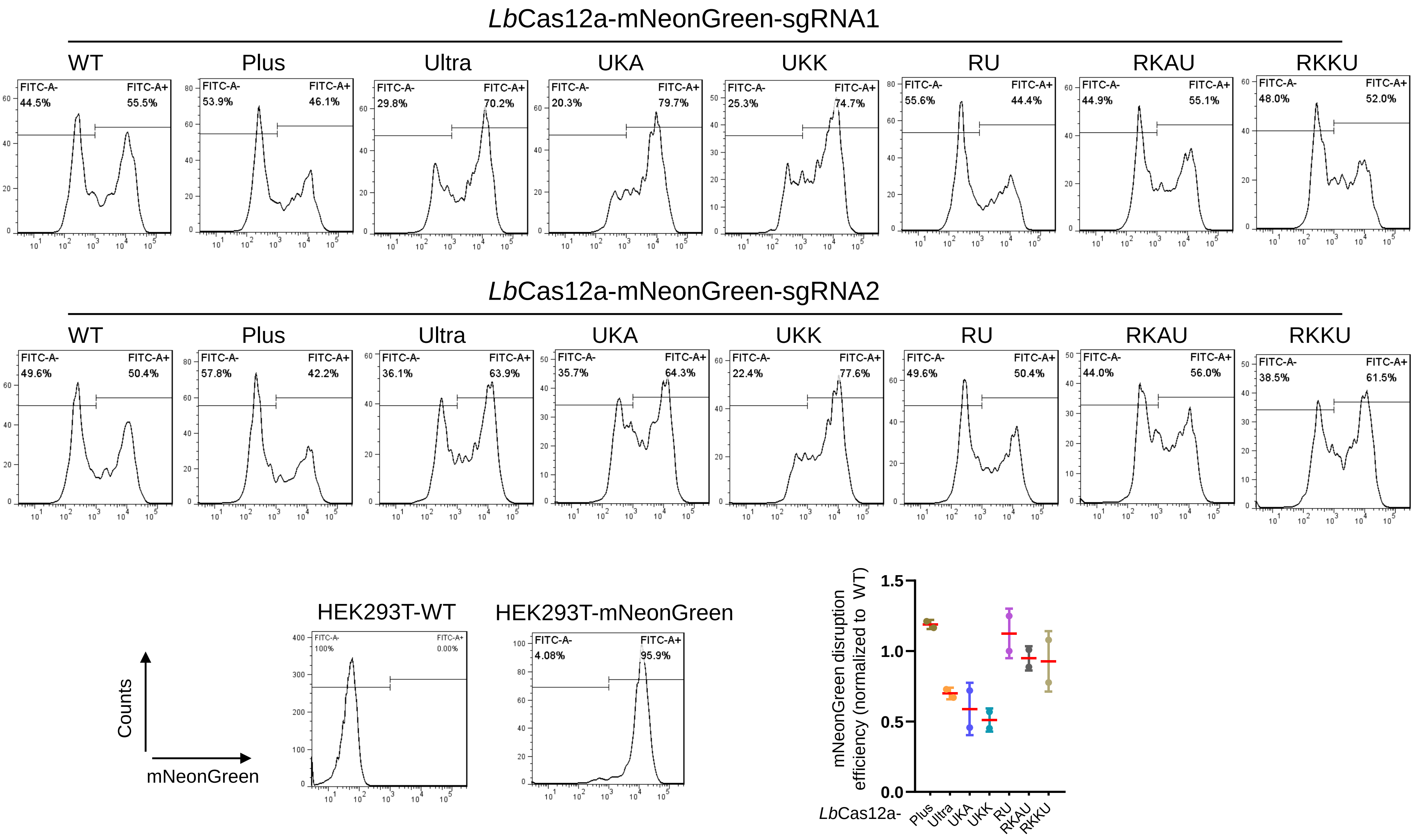

Figure S10. Editing efficiency analyses of *Lb*Cas12a-WT, -Plus, -ultra, -UKA, -UKK, -RU, -RKAU, and -RKKU

Figure S10. Editing efficiency analyses of *Lb*Cas12a-WT, -Plus, -ultra, -UKA, -UKK, -RU, -RKAU, and -RKKU. Flow cytometry detected the efficiency of disruption mNeonGreen by *Lb*Cas12a-WT and *As*Cas12a variants in HEK293T Knock-in mNeonGreen-PEST cell line. mNeonGree-sgRNA1/2, *Lb*Cas12a sgRNA targeting mNeonGreen site 1/2. n=2 independent experiments.
