# Supplementary material for: Engineered Cas12a-Plus nuclease enables gene editing with enhanced activity and specificity
Source: BMC Biol. 2022 Apr 25;20:91. doi: 10.1186/s12915-022-01296-1 (PMC9040236; doi:10.1186/s12915-022-01296-1)
Supplement: Supplementary file 2 — Additional file 2: Figure S1. Construction of AsCas12a variants and assessment of their specificity. Figure S2. Specificity assessment of AsCas12a-RKA. Figure S3. Specificity comparison of AsCas12a-WT, -RKA, -RKK, and -HF by Tag-seq. Figure S4. The Engineering of crRNA to improve efficiency. Figure S5. Editing of BRAFV600E with SpCas9 and AsCas12a nucleases. Figure S6. Detection the expression levels of LbCas12a nucleases. Figure S7. Specificity assessment of AsCas12a-WT, -Plus, -HF, enAsCas12a, -ultra, -UKA, -UKK, -RU, -RKAU, and -RKKU by Tag-seq. Figure S8. Specificity assessment of LbCas12a-WT, -Plus, -ultra, -UKA, -UKK, -RU, -RKAU, -RKKU by Tag-seq. Figure S9. Editing efficiency analyses of AsCas12a-WT, -Plus, -HF, enAsCas12a, -ultra, -UKA, -UKK, and -RU. Figure S10. Editing efficiency analyses of LbCas12a-WT, -Plus, -ultra, -UKA, -UKK, -RU, -RKAU, and -RKKU. [file 12915_2022_1296_MOESM2_ESM.pdf]

Supplementary Information

Supplementary Tables

Table S1. The engineering of the CRISPR-*As*Cas12a/*Lb*Cas12a systems

Cas12a type	Engineering	Efficiency	Specificity	Note	Targeting Ranges
*As*Cas12a;<br><br>*Lb*Cas12a	Cas-protein	↓	↑	(2017) *As*Cas12a-RR/A (S542R, K607R/K949A);<br>*As*Cas12a-RVR/A (S542R, K548V, N552R/K949A);<br>*Lb*Cas12a-RR (G532R, K595R);<br>*Lb*Cas12a-RVR (G532R, K538V, Y542R)[26]	↑
*Lb*Cas12a	Cas-protein	--	--	(2018, 2019) *Lb*Cas12a-RR(G532R, K595R); -RVR (G532R, K538V, Y542R) (tested in Plant)[41-43]	↑
*As*Cas12a;<br>*Lb*Cas12a	Cas-protein	--	↑	(2019) Synthetic chimeric Cas12a[44]	--
*As*Cas12a	Cas-protein	↑	↓	(2019) en*As*Cas12a (E174R, S542R, K548R);<br>en*As*Cas12a-HF1 (N282A, E174R, S542R, K548R)[27]	↑
*Lb*Cas12a	Cas-protein	↑	↓	(2020) *Lb*Cas12a-RRVR (G532R, K538V, Y542R, K595R);<br>imp*Lb*Cas12a (D156R, G532R, K538V, Y542R, K595R)[45]	↑
LbCas12a	Cas-protein	↑	Unknown	(2020) en*Lb*Cas12a (D156R, G532R, K538R); tt*Lb*Cas12a (D156R), (tested in Plant)[46]<br>(2020) *Lb*Cas12a (D156R), (tested in Drosophila)[47]	↑
d*Lb*Cas12a	Cas-protein	--	--	(2020) N256A, N260A, S348A, K514A, K881A, K897A[48]	Unknown
*Lb*Cas12a	Cas-protein	--	Unknown	(2020) *Lb*Cas12a-RSR (G532R/K538S/Y548R);<br>*Lb*Cas12a-RAR (G532R/K538A/Y548R)[49]	↑
*As*Cas12a	Cas-protein	↑	↓	(2021) *As*Cas12a ultra (M537R/F870L)[52]	↑
*Lb*Cas12a;<br>*As*Cas12a;<br>*Lb2*Cas12a	Cas-protein	--	↑	(2022) Lb-K538R;<br>As-K548R;<br>Lb2-K548R[53]	--
AsCas12a	Cas-protein<br>sgRNA	↑	Unknown<br>↓	(2020) opAsCas12a(E174R, S542R)[50];<br>(2020) open*As*Cas12a (E174R, S542R, K548R)[51]	Unknown<br>Unknown
*As*Cas12a;<br>*Lb*Cas12a	sgRNA	↑	--	(2017) sgRNA with chemical modifications[10]	Unknown
LbCas12a	sgRNA	--	--	(2017) A fusion sgRNA for both Cas9 and Cas12a[11]	Unknown
*Lb*Cas12a	sgRNA	↑	Unknown	(2018) tRNA-sgRNA intron[12]	Unknown
*As*Cas12a;<br>*Lb*Cas12a	sgRNA	↑	Unknown	(2018) ribozyme-processed crRNA[9]	Unknown
LbCas12a	sgRNA	--	Unknown	(2018) using short and long spacer crRNA[16]	Unknown
*Lb*Cas12a	sgRNA	↑	Unknown	(2018) truncated pre-tRNA-like sgRNA[7]	Unknown
*As*Cas12a	sgRNA	↑	Unknown	(2018) Extended sgRNA[8]	Unknown

*As*Cas12a; *Lb*Cas12a	sgRNA	↑	--	(2018) A U-rich crRNA sgRNA[6]	Unknown
*Lb*Cas12a; *Fno*Cas12a	sgRNA	↑	--	(2019) Full-DR-sgRNA[13]	Unknown
*As*Cas12a	sgRNA	--	↑	(2020) Chimeric DNA-RNA sgRNA[14]	Unknown
AsCas12a; LbCas12a	sgRNA and Cas-protein	↑	--	(2021) Chemical modification[15]	Unknown
*As*Cas12a; *Lb*Cas12a	Cas-protein	↑	↑	This study.<br>*As*Cas12a-Plus (E174R, R951K, R955A);<br>*Lb*Cas12a-Plus (D156R, R883K, R887A)	↑

Note: Unless special stated, the comparison is performed with wild-type Cas-nuclease. "--", Comparable. "↑", Enhance/Improve. "↓", Decrease. "Unknow", untested in the study.

Table S2. The engineering of the CRISPR-Cas12a (excluded *As*Cas12a and *Lb*Cas12a) systems

Cas12a type	Engineering	Efficiency	Specificity	Note	Targeting Ranges
*Fn*Cas12a	Cas-protein	--	Unknown	(2018) *Fn*Cas12a-RVR(N623R, K629R and N633R)<br>*Fn*Cas12a-RR(N623R, K687R) (tested in Plant)[42]	↑
*Fn*Cas12a	Cas-protein	↑	--	(2021) ea*Fn*Cas12a (Q125R)[54]	Unknown
MbCas12a;<br>FnCas12a	Cas-protein	--	Unknown	(2018) *Mb*Cas12a-RR (N576R, K637R);<br>*Mb*Cas12a-RVR (N576R, K582V, N586R);<br>*Fn*Cas12a-RR (N607R, K671R);<br>*Fn*Cas12a-RVR (N607R, K613V, N617R)[55]	↑
AaCas12a	--	--	↑	(2018) Specificity improved compared to *As*Cas12a and *SpCas9*[56]	Unknown
CeCas12a	Cas-protein	↑	--	(2020) Activity improved compared to *As*Cas12a and *Lb*Cas12a[57]	↑
PiCas12a	Cas-protein	↑	Unknown	(2020) *Pi*Cas12a (F604Y)[58]	--
FnCas12a	Cas-protein	↑	--	(2020) T5 Exonuclease Fusion to *Fn*Cas12a[59]	Unknown
*Lb2*Cas12a	Cas-protein	↑	--	(2021) Lb2-KY (Q571K, C1003Y)[60]	--
BsCas12a;<br>*Pr*Cas12a;<br>*Mb3*Cas12a	Cas-protein	↑	Unknown	(2021)*Bs*Cas12a-3Rv (K155R/N512R/K518R);<br>*Pr*Cas12a-3Rv (E162R/N519R/K525R);<br>*Mb3*Cas12a-3Rv (D180R/N581R/K587R)[61]	↑
*Mb2*Cas12a;<br>*Er*Cas12a;<br>*Lb5*Cas12a;<br>*Bs*Cas12a	Cas-protein	↑	Unknown	(2021) Mb2Cas12a-RVR(N563R, K569V, N573R);<br>Mb2Cas12a-RVRR(N563R, K569V, N573R, K625R)<br>ErCas12a-RVR(D529R, K535V, N539R);<br>Lb5Cas12a-RVR(N512R, K518V, N522R);<br>BsCas12a-RVR(N512R, K518V, N522R)[62]	↑

*Pr*Cas12a;<br>*Ar*Cas12a;<br>*Bs*Cas12a;<br>*Hk*Cas12a;<br>*Lp*Cas12a;<br>*Px*Cas12a	sgRNA	↑	--	(2019) optimized crRNA scaffolds[63]	--
TsCas12a;<br>*MlCas12a;*<br>*BoCas12a;*<br>*CMaCas12a*	others	--	--	(2021) The wild-type nuclease, tested in the plant[62]	--

Note: Unless special stated, the comparison is performed with the corresponding wild-type Cas-nuclease. “--", Comparable. “↑", Enhance/Improve/Widen. “↓", Decrease. “Unknow", untested in the study.

Table S3. The sgRNAs used in this study

Name	Sequence(5’-3’)	location (hg19)
Site 6	TTTNGGGTGATCAGACCCAACAGCAGG	chr3 - 129108493 129108519
CCR5-S1	TTTNTGGGCAACATGCTGGTCATCCTC	chr3 + 46414526 46414552
CCR5-S2	TTTNAAGCAAACACAGCATGGACGACA	chr3 - 46414780 46414806
CCR5-S3	TTTNGGATTCCCGAGTAGCAGATGACC	chr3 - 46415023 46415049
CD47-S1	TTTNAGAAATGAGAGAGAAACAAGATG	chr3 + 107805419 107805445
CD47-S2	TTTNATAAAACAAAATCTGTAGAATTC	chr3 - 107799148 107799174
CD47-S3	TTTNTCTCCCCTGCTTCCCACAACTAA	chr3 - 107805249 107805275
CD47-S4	TTTNAAAACAGAAAGAACATACTCATA	chr3 + 107798052 107798078
SIRPa-S1	TTTNAAAAGAGAGAGAGAGGGGGGAAA	chr20 - 1879740 1879766
SIRPa-S2	TTTNCTACAGTTACAACATTCCAACTG	chr20 + 1902671 1902697
SIRPa-S3	TTTNTGTCCGGGACACGTTTCCATTCT	chr20 - 1903070 1903096
SIRPa-S4	TTTNACCACTAAGCAGGCATCATTAAC	chr20 - 1905314 1905340
CXCR4-S1	TTTNAAAGTCACATCTTGGCTAACTCC	chr2 + 136873755 136873781
CXCR4-S2	TTTNCCACGGCATCAACTGCCCAGAAG	chr2 + 136873196 136873222
PD1-S1	TTTNCCCAGAATCTCCAGTTCTGAGTC	chr2 + 242799735 242799761
PD1-S2	TTTNAGGCCCAGCCAGCACTCTGGCCT	chr2 - 242792483 242792509
CTLA4-S1	TTTNTATGAAGTAGGTACTCAAAAGAT	chr2 + 204733604 204733630
RUNX1	TTTNGCTCCGAAGGTAAAAGAAATCAT	chr21 - 36421242 36421268
RUNX1-S1	TTTNTGTCTCTCTGCCCCCTCCCCACT	chr21 + 37371826 37371852
DNMT1-S3	TTTNCTGATGGTCCATGTCTGTTACTC	chr19 - 10244443 10244469
DNMT1-S7	TTTNGCTCAGCAGGCACCTGCCTCAGC	chr19 - 10133918 10133944
EMX1	TTTNTCCTCCGGTTCTGGAACCACACC	chr2 - 73160921 73160947
MYOD-S1	TTTNGGCTACTACGGATAAATAGCCCA	chr11 + 17741072 17741098
MYOD-S2	TTTNCCGCGGATACAGCAGTCGGGTGT	chr11 + 17740805 17740831
IL1RN-S1	TTTNCAGGAGGGTGACTCAGGCTAGCA	chr2 - 113875317 113875343
IL1RN-S3	TTTNTTAGAGCGTTGGGGACCTTGTCT	chr2 + 113875106 113875132
HBG-S4	TTTNAACTACAGGCCTCACTGGAGCTA	chr11 - 5271453 5271479/<br>chr11 - 5276381 5276407
HBG-S5	TTTNCCTTGTCAAGGCTATTGGTCAAG	chr11 + 5271181 5271207/<br>chr11 + 5276105 5276131
FANCF	TTTNGTCGGCATGGCCCCATTCGCACG	chr11 - 22647189 22647215

VEGFA-S1	TTTNCTGGAAAGAGCAGAAGAAAAAGG	chr6 - 43742342 43742368
BE-S3	TTTNTCCGTGTTCCTTGACTCTGGGCA	chr11 + 22647490 22647516
Site 16	TTTNGAAGCACATCAAGGACATTCTAA	chr21 + 38860226 38860252
Site 18	TTTNGTTTAAACACACCGGGTTAATAA	chr9 - 110247058 110247084
NPY1R	TTTNAAGCCTCGGGAAACTGCCCTGCC	chr4 - 164254002 164254028
CHN1	TTTNAGCTAAAACGCACACCGGCCCCG	chr2 - 175870275 175870301
SNX13	TTTNTCCTCTCCCCAGGGCGGGCCAAC	chr7 - 17980383 17980409
Cas12a-BRAF-WT	TTTNGTCTAGCTACAGTGAAATCTCGA	chr7 - 140453126 140453152
Cas12a-BRAF-Mut	TTTNGTCTAGCTACAGAGAAATCTCGA	chr7 - 140453126 140453152
Cas9-BRAF-WT	TAGCTACAGTGAAATCTCGANGG	chr7 - 140453123 140453145
Cas9-BRAF-Mut	TAGCTACAGAGAAATCTCGANGG	chr7 - 140453123 140453145
EGFP-g1	TTTNCGTCGCCGTCCAGCTCGACCAGG	
EGFP-g2	TTTNCTCAGGGCGGACTGGGTGCTCAG	
mNeonGreen-g1	TTTNCAGGCCGCTATGGTGGATGGCAG	
mNeonGreen-g2	TTTNGCGAAGGTGTAGGTGGTCCGGGC	
Activation sgRNA		
IL1RN-23bp	TTTNCAGGAGGGTGACTCAGGCTAGCA	chr2 - 113875317 113875343
MYOD-23bp	TTTNCCGCGGATACAGCAGTCGGGTGT	chr11 + 17740805 17740831
HBG-23bp	TTTNCCTTGTCAAGGCTATTGGTCAAG	chr11 + 5271181 5271207
Fgf21-23bp	TTTNTACCCAGACAGGCCCGCCCACGT	chr7 + 45615513 45615539
IL1RN-15bp	TTTNCAGGAGGGTGACTCA	chr2 - 113875325 113875343
MYOD-15bp	TTTNCCGCGGATACAGCAG	chr11 + 17740805 17740823
Non-canonical-PAM sgRNA		
ATTA	ATTAGAAGGAGATGCTCCTGTCTCCTG	chr7 + 117232231 117232257
CTTA	CTTATTGGGTCAGCTGTTAACATCAGT	chr19 - 10244479 10244505
GTTA	GTTATGAACCTGGGTGAAGTCCCAACA	chr2 - 73149328 73149354
TTTA	TTTAGGTCATCTGCTACTCGGGAATCC	chr3 + 46399672 46399694
TCTA	TCTATAGCCATTGAAGCGCAGCATGTG	chr11 + 22646901 22646927

Table S4. Primers for this study (including qPCR, T7E1/PAGE, Deep-seq)

Name	Sequence(5’-3’)
qPCR-IL1RN-F	GGAATCCATGGAGGGAAGAT
qPCR-IL1RN-R	TGTTCTCGCTCAGGTCAGTG
qPCR-MYOD-F	CCGACGGCATGATGGACTAC
qPCR-MYOD-R	AGGCAGTCTAGGCTCGACAC
qPCR-HBG-F	GCTGAGTGAACTGCACTGTGA
qPCR-HBG-R	GAATTCTTTGCCGAAATGGA
qPCR-Fgf21-F	CAAGACACTGAAGCCCACCT
qPCR-Fgf21-R	CACCCAGGATTTGAATGACC
qPCR-GAPDH-F	AGAAGGCTGGGGCTCATTTG
qPCR-GAPDH-R	AGGGGCCATCCACAGTCTTC
Gapdh-qPCR-F1	GGTGAAGGTCGGTGTGAAC
Gapdh-qPCR-R1	GAGTGGAGTCATACTGGAAC
PAGE/T7E1-DMNT1-site3-F	ACGCCTGCTCTAAGCACTTG

PAGE/T7E1-DMNT1-site3-R	TAGTTCTGCCCTCCCGTCAC
DMNT1-site3-Deep-Seq-F	atcgCTCTTTCCCTACACGACGCTCTTCCGATCTIndex1GTCACTCTGGGGAACACGC
DMNT1-site3-D Deep-Seq-R	ctctACTGGAGTTCAGACGTGTGCTCTTCCGATCTIndex2CCGTGAACGTTCCCTTAGCA
Site6-Deep-Seq-F	atcgCTCTTTCCCTACACGACGCTCTTCCGATCTIndex1CCACATCCTCACCACCTGTTT
Site6-Deep-Seq-R	ctctACTGGAGTTCAGACGTGTGCTCTTCCGATCTIndex2GGACAGGGTGGGTTACCTTG
FNACF-Deep-Seq-F	atcgCTCTTTCCCTACACGACGCTCTTCCGATCTIndex1TGCCTCCACTGGTTGTGCAG
FANCF-Deep-Seq-R	ctctACTGGAGTTCAGACGTGTGCTCTTCCGATCTIndex2CTTCTGGCGGTCTCAAGCA
MYOD-Deep-Seq-F	atcgCTCTTTCCCTACACGACGCTCTTCCGATCTIndex1CTACAGCCGCTCTACCCAT
MYOD-Deep-Seq-R	ctctACTGGAGTTCAGACGTGTGCTCTTCCGATCTIndex2TGCACTTGGCTCTCCGGCA
IL1RN-Deep-Seq-F	atcgCTCTTTCCCTACACGACGCTCTTCCGATCTIndex1TGACCTCCCATCTTACGCAG
IL1RN-Deep-Seq-R	ctctACTGGAGTTCAGACGTGTGCTCTTCCGATCTIndex2AGAGTACAAGGAGGAGCCCA

Table S5. Index information for deep-seq

Genes	sample		Index1	Index2
DNMT1-site3	AsCas12a-WT-On		AGAGTAGA	TAAGGCGA
	AsCas12a-WT-mm1		AGAGTAGA	CTAGTACG
	AsCas12a-WT-mm8		AGAGTAGA	TTCTGCCT
	AsCas12a-WT-mm9		AGAGTAGA	GCTCAGGA
	AsCas12a-A-On		TATCCTCT	TAAGGCGA
	AsCas12a-A-mm1		TATCCTCT	CTAGTACG
	AsCas12a-A-mm8		TATCCTCT	TTCTGCCT
	AsCas12a-A-mm9		TATCCTCT	GCTCAGGA
	AsCas12a-AKK-On		TAGATCGC	TAAGGCGA
	AsCas12a-AKK-mm1		TAGATCGC	CTAGTACG
	AsCas12a-AKK-mm8		TAGATCGC	TTCTGCCT
	AsCas12a-AKK-mm9		TAGATCGC	GCTCAGGA
	AsCas12a-AKA-On		CTCTCTAT	TAAGGCGA
	AsCas12a-AKA-mm1		CTCTCTAT	CTAGTACG
	AsCas12a-AKA-mm8		CTCTCTAT	TTCTGCCT
	AsCas12a-AKA-mm9		CTCTCTAT	GCTCAGGA
	AsCas12a-KK-On		GTAAGGAG	TAAGGCGA
	AsCas12a-KK-mm1		GTAAGGAG	CTAGTACG
	AsCas12a-KK-mm8		GTAAGGAG	TTCTGCCT
	AsCas12a-KKC-mm9		GTAAGGAG	GCTCAGGA
	AsCas12a-KA-On		ACTGCATA	TAAGGCGA
	AsCas12a-KA-mm1		ACTGCATA	CTAGTACG
	AsCas12a-KA-mm8		ACTGCATA	TTCTGCCT
	AsCas12a-KA-mm9		ACTGCATA	GCTCAGGA
	WT-crRNA (repeat 1)	AsCas12a-WT	GACATT	AGCGTA
		AsCas12a-KA	GTAAGG	AGCGTA
		AsCas12a-KK	ACTGCA	AGCGTA
		AsCas12a-RKA	CTCTCT	AGCGTA
		AsCas12a-RKK	AGAGTA	AGCGTA
		AsCas12a-HF	TAGATC	AGCGTA

Site6		Ctrl	AAGGAG	AGCGTA
	WT-crRNA (repeat 2)	AsCas12a-WT	GACATT	GCTCAG
		AsCas12a-KA	GTAAGG	GCTCAG
		AsCas12a-KK	ACTGCA	GCTCAG
		AsCas12a-RKA	CTCTCT	GCTCAG
		AsCas12a-RKK	AGAGTA	GCTCAG
		AsCas12a-HF	TAGATC	GCTCAG
		Ctrl	AAGGAG	GCTCAG
	U4AU6-crRNA (repeat 1)	AsCas12a-WT	GACATT	TCGCCT
		AsCas12a-KA	GTAAGG	TCGCCT
		AsCas12a-KK	ACTGCA	TCGCCT
		AsCas12a-RKA	CTCTCT	TCGCCT
		AsCas12a-RKK	AGAGTA	TCGCCT
		AsCas12a-HF	TAGATC	TCGCCT
		Ctrl	AAGGAG	TCGCCT
	U4AU6-crRNA (repeat 2)	AsCas12a-WT	GACATT	AGGAGT
		AsCas12a-KA	GTAAGG	AGGAGT
		AsCas12a-KK	ACTGCA	AGGAGT
		AsCas12a-RKA	CTCTCT	AGGAGT
		AsCas12a-RKK	AGAGTA	AGGAGT
		AsCas12a-HF	TAGATC	AGGAGT
		Ctrl	AAGGAG	AGGAGT
	tRNA-crRNA (repeat 1)	AsCas12a-WT	GACATT	CTAGTA
		AsCas12a-KA	GTAAGG	CTAGTA
		AsCas12a-KK	ACTGCA	CTAGTA
		AsCas12a-RKA	CTCTCT	CTAGTA
		AsCas12a-RKK	AGAGTA	CTAGTA
		AsCas12a-HF	TAGATC	CTAGTA
		Ctrl	AAGGAG	CTAGTA
	tRNA-crRNA (repeat 2)	AsCas12a-WT	GACATT	CATGCC
		AsCas12a-KA	GTAAGG	CATGCC
		AsCas12a-KK	ACTGCA	CATGCC
		AsCas12a-RKA	CTCTCT	CATGCC
		AsCas12a-RKK	AGAGTA	CATGCC
		AsCas12a-HF	TAGATC	CATGCC
		Ctrl	AAGGAG	CATGCC
	WT-crRNA (repeat 1)	AsCas12a-WT	TATCCT	TTCTGC
		AsCas12a-KA	GTAAGG	TTCTGC
		AsCas12a-KK	ACTGCA	TTCTGC
		AsCas12a-RKA	CTCTCT	TTCTGC
		AsCas12a-RKK	AGAGTA	TTCTGC
		AsCas12a-HF	TAGATC	TTCTGC
		Ctrl	AAGGAG	TTCTGC
	WT-crRNA (repeat 2)	AsCas12a-WT	TATCCT	GCTCAG
		AsCas12a-KA	GTAAGG	GCTCAG
		AsCas12a-KK	ACTGCA	GCTCAG
		AsCas12a-RKA	CTCTCT	GCTCAG
		AsCas12a-RKK	AGAGTA	GCTCAG
		AsCas12a-HF	TAGATC	GCTCAG

FANCF		Ctrl	AAGGAG	GCTCAG
	U4AU6-crRNA (repeat 1)	AsCas12a-WT	TATCCT	TCGCCT
		AsCas12a-KA	GTAAGG	TCGCCT
		AsCas12a-KK	ACTGCA	TCGCCT
		AsCas12a-RKA	CTCTCT	TCGCCT
		AsCas12a-RKK	AGAGTA	TCGCCT
		AsCas12a-HF	TAGATC	TCGCCT
		Ctrl	AAGGAG	TCGCCT
	U4AU6-crRNA (repeat 2)	AsCas12a-WT	TATCCT	AGGAGT
		AsCas12a-KA	GTAAGG	AGGAGT
		AsCas12a-KK	ACTGCA	AGGAGT
		AsCas12a-RKA	CTCTCT	AGGAGT
		AsCas12a-RKK	AGAGTA	AGGAGT
		AsCas12a-HF	TAGATC	AGGAGT
		Ctrl	AAGGAG	AGGAGT
	tRNA-crRNA (repeat 1)	AsCas12a-WT	TATCCT	CTAGTA
		AsCas12a-KA	GTAAGG	CTAGTA
		AsCas12a-KK	ACTGCA	CTAGTA
		AsCas12a-RKA	CTCTCT	CTAGTA
		AsCas12a-RKK	AGAGTA	CTAGTA
		AsCas12a-HF	TAGATC	CTAGTA
		Ctrl	AAGGAG	CTAGTA
	tRNA-crRNA (repeat 2)	AsCas12a-WT	TATCCT	CATGCC
		AsCas12a-KA	GTAAGG	CATGCC
		AsCas12a-KK	ACTGCA	CATGCC
		AsCas12a-RKA	CTCTCT	CATGCC
		AsCas12a-RKK	AGAGTA	CATGCC
		AsCas12a-HF	TAGATC	CATGCC
		Ctrl	AAGGAG	CATGCC
MYOD (23bp and 15 bp)	Repeat 1	WT-VPR	GACATT	AGCGTA
		RKA-VPR	TAGATC	AGCGTA
		HF-VPR	CTCTCT	AGCGTA
		Ctrl	AGAGTA	AGCGTA
	Repeat 2	WT-VPR	GACATT	TCGCCT
		RKA-VPR	TAGATC	TCGCCT
		HF-VPR	CTCTCT	TCGCCT
		Ctrl	AGAGTA	TCGCCT
	Repeat 3	WT-VPR	GACATT	CTAGTA
		RKA-VPR	TAGATC	CTAGTA
		HF-VPR	CTCTCT	CTAGTA
		Ctrl	AGAGTA	CTAGTA
IL1RN (23bp and 15 bp)	Repeat 1	WT-VPR	GACATT	AGCGTA
		RKA-VPR	TAGATC	AGCGTA
		HF-VPR	CTCTCT	AGCGTA
		Ctrl	AGAGTA	AGCGTA
	Repeat 2	WT-VPR	GACATT	TCGCCT
		RKA-VPR	TAGATC	TCGCCT
		HF-VPR	CTCTCT	TCGCCT
		Ctrl	AGAGTA	TCGCCT

	Repeat 3	WT-VPR<br>RKA-VPR<br>HF-VPR<br>Ctrl	GACATT<br>TAGATC<br>CTCTCT<br>AGAGTA	CTAGTA<br>CTAGTA<br>CTAGTA<br>CTAGTA

Supplementary sequences: DNA and Amino acid sequences used in this study

- Amplicons for high-throughput sequencing analysis

DMNT1-site3:
GTCACTCTGGGGAACACGCCCGGTGTCACGCCACTTGACAGGCGAGTAACAGACATGGACCATCAGGAAA
CATTAACGTACTGATGTTAACAGCTGACCCAATAAGTGGCAGAGTGCTAAGGGAACGTTCACGG
FANCF:
TGCCTCCACTGGTTGTGCAGCCGCCGCTCCAGAGCCGTGCGAATGGGGCCATGCCGACCAAAGCGCCGAT
GGATGTGGCGCAGGTAGCGCGCCCACTGCAAGGCCCGGCGCACGGTGGCGGGGTCCCAGGTGCTGACG
TAGGTAGTGCTTGAGACCGCCAGAAG
Site 6:
CCACATCCTCACCACCTGTTTGTTGCAGCCTCCACACTTGTGATAGCCCCCTGGTCCCATTTTGTCTCTCAAA
CTGTCTTTCTCATTCCTTGGACTCCACTGGACTTCGTCGCCCCCATGACCTGCTGTTGGGTCTGATCACCCC
AAAAGAAATGAGGTTTGAGTATGTTAGAGTATGCCAAGGTAACCCACCCTGTCC
MYOD:
CTACAGCCGCTCTACCCATGGGTCCCCCACAAATCAGGGGACAGAGGAGTATTGAAAGTCAGCTCAGAGGT
GAGCGCGCGCAGCCAGCGTTTCCCGCGGATACAGCAGTCGGGTGTTGGAGAGGTTTGGAAAGGGCGTGC
CGGAGAGCCAAGTGCA
IL1RN:
TGACCTCCCATCTTACGCAGATAAGAACCAGTTTGGTTTCTGCTAGCCTGAGTCACCCTCCTGGAAACTGGG
CCTGCTTGGCATCAAGTCAGCCATCAGCCGGCCCATCTCCTCATGCTGGCCAACCCTCTGTGAGTGTGTGG
GAGGGGAGGCTGGGCTCCTCCTTGTACTCT

- Expression of the pe-tRNA

Red: EF1a core promoter, blue: tRNA, orange: AsCpf1-crRNA scaffold, violet: SV40 polyA
GGGCAGAGCGCACATCGCCCACAGTCCCCGAGAAGTTGGGGGGAGGGGTCGGCAATTGATCCGGTGCCT
AGAGAAGGTGGCGCGGGGTAAACTGGGAAAGTGATGTCGTGTACTGGCTCCGCCTTTTTCCCGAGGGTGG
GGGAGAACCGTATATAAGTGCAGTAGTCGCCGTGAACGTTCTTTTTCGCAACGGGTTTGCCGCCAGAACACA
GCCAGTGGTCTAGTGGTAGAATAGTACCCTGCCACGGTACAGACCCGGGTTCGATTCCCGGCTGGAAATAAT
TTCTACTCTTGTAGAT/23bp-sgRNA/TTTTTTGGATCCACTAGTTCTAGAAACTTGTTTATTGCAGCTTATAATGG
TTACAAATAAAGCAATAGCATCACAAATTTCACAAATAAAGCATTTTTTTCACTGCATTCTAGTTGTGGTTTGTC
CAAACTCATCAATGTATCTTA

- Expression of the T4AT6-crRNA

Red: U6 promoter, orange: AsCpf1-crRNA scaffold, violet: T4AT6 motif
AAGGTCGGGCAGGAAGAGGGCCTATTTCCCATGATTCCTTCATATTTGCATATACGATACAAGGCTGTTAGAG
AGATAATTAGAATTAATTTGACTGTAAACACAAAGATATTAGTACAAAATACGTGACGTAGAAAGTAATAATTTCT
TGGGTAGTTTGCAGTTTTAAAATTATGTTTTAAAATGGACTATCATATGCTTACCGTAACTTGAAAGTATTTCGAT
TTCTTGGCTTTATATATCTTGTGGAAAGGACGAAACACCGTAATTTCTACTCTTGTAGAT/20bp-sgRNA/TTTTAT
TTTTT

- Expression of crRNA array

Red: U6 promoter, orange: AsCpf1-crRNA scaffold, sgRNA targeted to DNMT1-S7, EMX1, CTLA4-S1, CCR5-S1, SIRPa-S3, RUNX1-S1
AAGGTCGGGCAGGAAGAGGGCCTATTTCCCATGATTCCTTCATATTTGCATATACGATACAAGGCTGTTAGAG
AGATAATTAGAATTAATTTGACTGTAAACACAAAGATATTAGTACAAAATACGTGACGTAGAAAGTAATAATTTCT
TGGGTAGTTTGCAGTTTTAAAATTATGTTTTAAAATGGACTATCATATGCTTACCGTAACTTGAAAGTATTTCGAT

TTCTTGGCTTTATATATCTTGTGGAAAGGACGAAACACCGTAATTTCTACTCTTGTAGATGCTCAGCAGGCACC
TGCCTCAGCAATTTCTACTCTTGTAGATTCCTCCGGTTCTGGAACCACACCAATTTCTACTCTTGTAGATTATG
AAGTAGGTACTCAAAAGATAATTTCTACTCTTGTAGATTGGGCAACATGCTGGTCATCCTCAATTTCTACTCTT
GTAGATTGTCCGGGACACGTTTCCATTCTAATTTCTACTCTTGTAGATTGTCTCTCTGCCCCCTCCCCACTTTT
TTTTG

- dWT-VPR: pCAG-dAsCpf1-WT (D908A)-NLS-3xHA-VPR

Human codon optimized Acidaminococcus sp. BV3L6 Cpf1 (AsCpf1) is black, mutant site highlighted with red, nucleoplasmin NLS with blue, 3xHA with orange, VPR with violet

ATGACACAGTTCGAGGGCTTTACCAACCTGTATCAGGTGAGCAAGACACTGCGGTTTGAGCTGATCCCACA
GGGCAAGACCCTGAAGCACATCCAGGAGCAGGGCTTCATCGAGGAGGACAAGGCCCGCAATGATCACTAC
AAGGAGCTGAAGCCCATCATCGATCGGATCTACAAGACCTATGCCGACCAGTGCCTGCAGCTGGTGCAGCT
GGATTGGGAGAACCTGAGCGCCGCCATCGACTCCTATAGAAAGGAGAAAACCGAGGAGACAAGGAACGCC
CTGATCGAGGAGCAGGCCACATATCGCAATGCCATCCACGACTACTTCATCGGCCGGACAGACAACCTGAC
CGATGCCATCAATAAGAGACACGCCGAGATCTACAAGGGCCTGTTCAAGGCCGAGCTGTTTAATGGCAAGGT
GCTGAAGCAGCTGGGCACCGTGACCACAACCGAGCACGAGAACGCCCTGCTGCGGAGCTTCGACAAGTTT
ACAACCTACTTCTCCGGCTTTTATGAGAACAGGAAGAACGTGTTCAGCGCCGAGGATATCAGCACAGCCATC
CCACACCGCATCGTGCAGGACAACTTCCCCAAGTTTAAGGAGAATTGTCACATCTTCACACGCCTGATCACC
GCCGTGCCCAGCCTGCGGGAGCACTTTGAGAACGTGAAGAAGGCCATCGGCATCTTCGTGAGCACCTCCA
TCGAGGAGGTGTTTTCCTTCCCTTTTTATAACCAGCTGCTGACACAGACCCAGATCGACCTGTATAACCAGCT
GCTGGGAGGAATCTCTCGGGAGGCAGGCACCGAGAAGATCAAGGGCCTGAACGAGGTGCTGAATCTGGCC
ATCCAGAAGAATGATGAGACAGCCCACATCATCGCCTCCCTGCCACACAGATTCATCCCCCTGTTTAAGCAG
ATCCTGTCCGATAGGAACACCCTGTCTTTCATCCTGGAGGAGTTTAAGAGCGACGAGGAAGTGATCCAGTCC
TTCTGCAAGTACAAGACACTGCTGAGAAACGAGAACGTGCTGGAGACAGCCGAGGCCCTGTTTAACGAGCT
GAACAGCATCGACCTGACACACATCTTCATCAGCCACAAGAAGCTGGAGACAATCAGCAGCGCCCTGTGCG
ACCACTGGGATACACTGAGGAATGCCCTGTATGAGCGGAGAATCTCCGAGCTGACAGGCAAGATCACCAAG
TCTGCCAAGGAGAAGGTGCAGCGCAGCCTGAAGCACGAGGATATCAACCTGCAGGAGATCATCTCTGCCGC
AGGCAAGGAGCTGAGCGAGGCCTTCAAGCAGAAAACCAGCGAGATCCTGTCCCACGCACACGCCGCCCTG
GATCAGCCACTGCCTACAACCCTGAAGAAGCAGGAGGAGAAGGAGATCCTGAAGTCTCAGCTGGACAGCCT
GCTGGGCCTGTACCACCTGCTGGACTGGTTTGCCGTGGATGAGTCCAACGAGGTGGACCCCGAGTTCTCT
GCCCGGCTGACCGGCATCAAGCTGGAGATGGAGCCTTCTCTGAGCTTCTACAACAAGGCCAGAAATTATGC
CACCAAGAAGCCCTACTCCGTGGAGAAGTTCAAGCTGAACTTTCAGATGCCTACACTGGCCTCTGGCTGGG
ACGTGAATAAGGAGAAGAACAATGGCGCCATCCTGTTTGTGAAGAACGGCCTGTACTATCTGGGCATCATGC
CAAAGCAGAAGGGCAGGTATAAGGCCCTGAGCTTCGAGCCCACAGAGAAAACCAGCGAGGGCTTTGATAAG
ATGTACTATGACTACTTCCCTGATGCCGCCAAGATGATCCCAAAGTGCAGCACCCAGCTGAAGGCCGTGACA
GCCCACTTTCAGACCCACACAACCCCCATCCTGCTGTCCAACAATTTCATCGAGCCTCTGGAGATCACAAAG
GAGATCTACGACCTGAACAATCCTGAGAAGGAGCCAAAGAAGTTTCAGACAGCCTACGCCAAGAAAACCGG
CGACCAGAAGGGCTACAGAGAGGCCCTGTGCAAGTGGATCGACTTCACAAGGGATTTTCTGTCCAAGTATA
CCAAGACAACCTCTATCGATCTGTCTAGCCTGCGGCCATCCTCTCAGTATAAGGACCTGGGCGAGTACTATG
CCGAGCTGAATCCCCTGCTGTACCACATCAGCTTCCAGAGAATCGCCGAGAAGGAGATCATGGATGCCGTG
GAGACAGGCAAGCTGTACCTGTTCCAGATCTATAACAAGGACTTTGCCAAGGGCCACCACGGCAAGCCTAAT
CTGCACACACTGTATTGGACCGGCCTGTTTTCTCCAGAGAACCTGGCCAAGACAAGCATCAAGCTGAATGG
CCAGGCCGAGCTGTTCTACCGCCCTAAGTCCAGGATGAAGAGGATGGCACACCGGCTGGGAGAGAAGATG
CTGAACAAGAAGCTGAAGGATCAGAAAACCCCAATCCCCGACACCCTGTACCAGGAGCTGTACGACTATGT
GAATCACAGACTGTCCCACGACCTGTCTGATGAGGCCAGGGCCCTGCTGCCCAACGTGATCACCAAGGAG
GTGTCTCACGAGATCATCAAGGATAGGCGCTTTACCAGCGACAAGTTCTTTTTCCACGTGCCTATCACACTGA
ACTATCAGGCCGCCAATTCCCCATCTAAGTTCAACCAGAGGGTGAATGCCTACCTGAAGGAGCACCCCGAG
ACACCTATCATCGGCATCGCCCGGGGCGAGAGAAACCTGATCTATATCACAGTGATCGACTCCACCGGCAAG
ATCCTGGAGCAGCGGAGCCTGAACACCATCCAGCAGTTTGATTACCAGAAGAAGCTGGACAACAGGGAGAA
GGAGAGGGTGGCAGCAAGGCAGGCCTGGTCTGTGGTGGGCACAATCAAGGATCTGAAGCAGGGCTATCTG

AGCCAGGTCATCCACGAGATCGTGGACCTGATGATCCACTACCAGGCCGTGGTGGTGCTGGAGAACCTGAA
TTTCGGCTTTAAGAGCAAGAGGACCGGCATCGCCGAGAAGGCCGTGTACCAGCAGTTCGAGAAGATGCTGA
TCGATAAGCTGAATTGCCTGGTGCTGAAGGACTATCCAGCAGAGAAAGTGGGAGGCGTGCTGAACCCATAC
CAGCTGACAGACCAGTTCACCTCCTTTGCCAAGATGGGCACCCAGTCTGGCTTCCTGTTTTACGTGCCTGC
CCCATATACATCTAAGATCGATCCCCTGACCGGCTTCGTGGACCCCTTCGTGTGGAAAACCATCAAGAATCAC
GAGAGCCGCAAGCACTTCCTGGAGGGCTTCGACTTTCTGCACTACGACGTGAAAACCGGCGACTTCATCCT
GCACTTTAAGATGAACAGAAATCTGTCCTTCCAGAGGGGCCTGCCCGGCTTTATGCCTGCATGGGATATCGT
GTTCGAGAAGAACGAGACACAGTTTGACGCCAAGGGCACCCCTTTCATCGCCGGCAAGAGAATCGTGCCA
GTGATCGAGAATCACAGATTCACCGGCAGATACCGGGACCTGTATCCTGCCAACGAGCTGATCGCCCTGCT
GGAGGAGAAGGGCATCGTGTTCAGGGATGGCTCCAACATCCTGCCAAAGCTGCTGGAGAATGACGATTCTC
ACGCCATCGACACCATGGTGGCCCTGATCCGCAGCGTGCTGCAGATGCGGAACTCCAATGCCGCCACAGG
CGAGGACTATATCAACAGCCCCGTGCGCGATCTGAATGGCGTGTGCTTCGACTCCCGGTTTCAGAACCCAG
AGTGGCCCATGGACGCCGATGCCAATGGCGCCTACCACATCGCCCTGAAGGGCCAGCTGCTGCTGAATCA
CCTGAAGGAGAGCAAGGATCTGAAGCTGCAGAACGGCATCTCCAATCAGGACTGGCTGGCCTACATCCAGG
AGCTGCGCAACAAAAGGCCGGCGGCCACGAAAAAGGCCGGCCAGGCAAAAAAGAAAAAGGGATCCTACCC
ATACGATGTTCCAGATTACGCTTATCCCTACGACGTGCCTGATTATGCATACCCATACGATGTCCCCGACTATG
CCCTCGAGAGCACCGGTGATGCTTTAGACGATTTTGACTTAGATATGCTTGGTTCAGACGCGTTAGACGACT
TCGACCTAGACATGTTAGGCTCAGATGCATTGGACGACTTCGATTTAGATATGTTGGGCTCCGATGCCCTAGA
TGACTTTGATCTAGATATGCTAGGAGGAAGCGGAGGAGGAGGTAGCGGACCTAAGAAAAAGAGGAAGGTGG
CGGCCGCTGGATCCCCTTCAGGGCAGATCAGCAACCAGGCCCTGGCTCTGGCCCCTAGCTCCGCTCCAGT
GCTGGCCCAGACTATGGTGCCCTCTAGTGCTATGGTGCCTCTGGCCCAGCCACCTGCTCCAGCCCCTGTGC
TGACCCCAGGACCACCCCAGTCACTGAGCGCTCCAGTGCCCAAGTCTACACAGGCCGGCGAGGGGACTCT
GAGTGAAGCTCTGCTGCACCTGCAGTTCGACGCTGATGAGGACCTGGGAGCTCTGCTGGGGAACAGCACC
GATCCCGGAGTGTTCACAGATCTGGCCTCCGTGGACAACTCTGAGTTTCAGCAGCTGCTGAATCAGGGCGT
GTCCATGTCTCATAGTACAGCCGAACCAATGCTGATGGAGTACCCCGAAGCCATTACCCGGCTGGTGACCG
GCAGCCAGCGGCCCCCCGACCCCGCTCCAACTCCCCTGGGAACCAGCGGCCTGCCTAATGGGCTGTCCG
GAGATGAAGACTTCTCAAGCATCGCTGATATGGACTTTAGTGCCCTGCTGTCACAGATTTCCTCTAGTGGGTC
TGGCAGCGGCAGCGACCTTTCCCATCCGCCCCCAAGGGGCCATCTGGATGAGCTGACAACCACACTTGAG
TCCATGACCGAGGATCTGAACCTGGACTCACCCCTGACCCCGGAATTGAACGAGATTCTGGATACCTTCCTG
AACGACGAGTGCCTCTTGCATGCCATGCATATCAGCACAGGACTGTCCATCTTCGACACATCTCTGTTTAAGT
AA

- dRKA-VPR: pCAG-dAsCpf1-RKA(E174R/R951K/R955A/D908A)-NLS-3xHA-VPR

Human codon optimized Acidaminococcus sp. BV3L6 Cpf1 (AsCpf1) is black, mutant site highlighted with red, nucleoplasmin NLS with blue, 3xHA with orange, VPR with violet

ATGACACAGTTCGAGGGCTTTACCAACCTGTATCAGGTGAGCAAGACACTGCGGTTTGAGCTGATCCCACA
GGGCAAGACCCTGAAGCACATCCAGGAGCAGGGCTTCATCGAGGAGGACAAGGCCCGCAATGATCACTAC
AAGGAGCTGAAGCCCATCATCGATCGGATCTACAAGACCTATGCCGACCAGTGCCTGCAGCTGGTGCAGCT
GGATTGGGAGAACCTGAGCGCCGCCATCGACTCCTATAGAAAGGAGAAAACCGAGGAGACAAGGAACGCC
CTGATCGAGGAGCAGGCCACATATCGCAATGCCATCCACGACTACTTCATCGGCCGGACAGACAACCTGAC
CGATGCCATCAATAAGAGACACGCCGAGATCTACAAGGGCCTGTTCAAGGCCGAGCTGTTTAATGGCAAGGT
GCTGAAGCAGCTGGGCACCGTGACCACAACCGAGCACGAGAACGCCCTGCTGCGGAGCTTCGACAAGTTT
ACAACCTACTTCTCCGGCTTTTATAGAAACAGGAAGAACGTGTTCAGCGCCGAGGACATTAGCACAGCCATC
CCACACCGCATCGTGCAGGACAACTTCCCCAAGTTTAAGGAGAATTGTCACATCTTCACACGCCTGATCACC
GCCGTGCCCAGCCTGCGGGAGCACTTTGAGAACGTGAAGAAGGCCATCGGCATCTTCGTGAGCACCTCCA
TCGAGGAGGTGTTTTCCTTCCCTTTTTATAACCAGCTGCTGACACAGACCCAGATCGACCTGTATAACCAGCT
GCTGGGAGGAATCTCTCGGGAGGCAGGCACCGAGAAGATCAAGGGCCTGAACGAGGTGCTGAATCTGGCC
ATCCAGAAGAATGATGAGACAGCCCACATCATCGCCTCCCTGCCACACAGATTCATCCCCCTGTTTAAGCAG
ATCCTGTCCGATAGGAACACCCTGTCTTTCATCCTGGAGGAGTTTAAGAGCGACGAGGAAGTGATCCAGTCC
TTCTGCAAGTACAAGACACTGCTGAGAAACGAGAACGTGCTGGAGACAGCCGAGGCCCTGTTTAACGAGCT

GAACAGCATCGACCTGACACACATCTTCATCAGCCACAAGAAGCTGGAGACAATCAGCAGCGCCCTGTGCG
ACCACTGGGATACACTGAGGAATGCCCTGTATGAGCGGAGAATCTCCGAGCTGACAGGCAAGATCACCAAG
TCTGCCAAGGAGAAGGTGCAGCGCAGCCTGAAGCACGAGGATATCAACCTGCAGGAGATCATCTCTGCCGC
AGGCAAGGAGCTGAGCGAGGCCTTCAAGCAGAAAACCAGCGAGATCCTGTCCCACGCACACGCCGCCCTG
GATCAGCCACTGCCTACAACCCTGAAGAAGCAGGAGGAGAAGGAGATCCTGAAGTCTCAGCTGGACAGCCT
GCTGGGCCTGTACCACCTGCTGGACTGGTTTGCCGTGGATGAGTCCAACGAGGTGGACCCCGAGTTCTCT
GCCCGGCTGACCGGCATCAAGCTGGAGATGGAGCCTTCTCTGAGCTTCTACAACAAGGCCAGAAATTATGC
CACCAAGAAGCCCTACTCCGTGGAGAAGTTCAAGCTGAACTTTCAGATGCCTACACTGGCCTCTGGCTGGG
ACGTGAATAAGGAGAAGAACAATGGCGCCATCCTGTTTGTGAAGAACGGCCTGTACTATCTGGGCATCATGC
CAAAGCAGAAGGGCAGGTATAAGGCCCTGAGCTTCGAGCCCACAGAGAAAACCAGCGAGGGCTTTGATAAG
ATGTACTATGACTACTTCCCTGATGCCGCCAAGATGATCCCAAAGTGCAGCACCCAGCTGAAGGCCGTGACA
GCCCACTTTCAGACCCACACAACCCCCATCCTGCTGTCCAACAATTTCATCGAGCCTCTGGAGATCACAAAG
GAGATCTACGACCTGAACAATCCTGAGAAGGAGCCAAAGAAGTTTCAGACAGCCTACGCCAAGAAAACCGG
CGACCAGAAGGGCTACAGAGAGGCCCTGTGCAAGTGGATCGACTTCACAAGGGATTTTCTGTCCAAGTATA
CCAAGACAACCTCTATCGATCTGTCTAGCCTGCGGCCATCCTCTCAGTATAAGGACCTGGGCGAGTACTATG
CCGAGCTGAATCCCCTGCTGTACCACATCAGCTTCCAGAGAATCGCCGAGAAGGAGATCATGGATGCCGTG
GAGACAGGCAAGCTGTACCTGTTCCAGATCTATAACAAGGACTTTGCCAAGGGCCACCACGGCAAGCCTAAT
CTGCACACACTGTATTGGACCGGCCTGTTTTCTCCAGAGAACCTGGCCAAGACAAGCATCAAGCTGAATGG
CCAGGCCGAGCTGTTCTACCGCCCTAAGTCCAGGATGAAGAGGATGGCACACCGGCTGGGAGAGAAGATG
CTGAACAAGAAGCTGAAGGATCAGAAAACCCCAATCCCCGACACCCTGTACCAGGAGCTGTACGACTATGT
GAATCACAGACTGTCCCACGACCTGTCTGATGAGGCCAGGGCCCTGCTGCCCAACGTGATCACCAAGGAG
GTGTCTCACGAGATCATCAAGGATAGGCGCTTTACCAGCGACAAGTTCTTTTTCCACGTGCCTATCACACTGA
ACTATCAGGCCGCCAATTCCCCATCTAAGTTCAACCAGAGGGTGAATGCCTACCTGAAGGAGCACCCCGAG
ACACCTATCATCGGCATCGCCCGGGGCGAGAGAAACCTGATCTATATCACAGTGATCGACTCCACCGGCAAG
ATCCTGGAGCAGCGGAGCCTGAACACCATCCAGCAGTTTGATTACCAGAAGAAGCTGGACAACAGGGAGAA
GGAGAAGGTGGCGGCCGCTCAGGCCTGGTCTGTGGTGGGCACAATCAAGGATCTGAAGCAGGGCTATCTG
AGCCAGGTCATCCACGAGATCGTGGACCTGATGATCCACTACCAGGCCGTGGTGGTGCTGGAGAACCTGAA
TTTCGGCTTTAAGAGCAAGAGGACCGGCATCGCCGAGAAGGCCGTGTACCAGCAGTTCGAGAAGATGCTGA
TCGATAAGCTGAATTGCCTGGTGCTGAAGGACTATCCAGCAGAGAAAGTGGGAGGCGTGCTGAACCCATAC
CAGCTGACAGACCAGTTCACCTCCTTTGCCAAGATGGGCACCCAGTCTGGCTTCCTGTTTTACGTGCCTGC
CCCATATACATCTAAGATCGATCCCCTGACCGGCTTCGTGGACCCCTTCGTGTGGAAAACCATCAAGAATCAC
GAGAGCCGCAAGCACTTCCTGGAGGGCTTCGACTTTCTGCACTACGACGTGAAAACCGGCGACTTCATCCT
GCACTTTAAGATGAACAGAAATCTGTCCTTCCAGAGGGGCCTGCCCGGCTTTATGCCTGCATGGGATATCGT
GTTCGAGAAGAACGAGACACAGTTTGACGCCAAGGGCACCCCTTTCATCGCCGGCAAGAGAATCGTGCCA
GTGATCGAGAATCACAGATTCACCGGCAGATACCGGGACCTGTATCCTGCCAACGAGCTGATCGCCCTGCT
GGAGGAGAAGGGCATCGTGTTCAGGGATGGCTCCAACATCCTGCCAAAGCTGCTGGAGAATGACGATTCTC
ACGCCATCGACACCATGGTGGCCCTGATCCGCAGCGTGCTGCAGATGCGGAACTCCAATGCCGCCACAGG
CGAGGACTATATCAACAGCCCCGTGCGCGATCTGAATGGCGTGTGCTTCGACTCCCGGTTTCAGAACCCAG
AGTGGCCCATGGACGCCGATGCCAATGGCGCCTACCACATCGCCCTGAAGGGCCAGCTGCTGCTGAATCA
CCTGAAGGAGAGCAAGGATCTGAAGCTGCAGAACGGCATCTCCAATCAGGACTGGCTGGCCTACATCCAGG
AGCTGCGCAACAAAAAGGCCGGCGGCCACGAAAAAGGCCGGCCAGGCAAAAAAGAAAAAGGGATCCTACC
CATACGATGTTCCAGATTACGCTTATCCCTACGACGTGCCTGATTATGCATACCCATACGATGTCCCCGACTAT
GCCCTCGAGAGCACCGGTGATGCTTTAGACGATTTTGACTTAGATATGCTTGGTTCAGACGCGTTAGACGAC
TTCGACCTAGACATGTTAGGCTCAGATGCATTGGACGACTTCGATTTAGATATGTTGGGCTCCGATGCCCTAG
ATGACTTTGATCTAGATATGCTAGGAGGAAGCGGAGGAGGAGGTAGCGGACCTAAGAAAAAGAGGAAGGTG
GCGGCCGCTGGATCCCCTTCAGGGCAGATCAGCAACCAGGCCCTGGCTCTGGCCCCTAGCTCCGCTCCAG
TGCTGGCCCAGACTATGGTGCCCTCTAGTGCTATGGTGCCTCTGGCCCAGCCACCTGCTCCAGCCCCTGTG
CTGACCCCAGGACCACCCCAGTCACTGAGCGCTCCAGTGCCCAAGTCTACACAGGCCGGCGAGGGGACTC
TGAGTGAAGCTCTGCTGCACCTGCAGTTCGACGCTGATGAGGACCTGGGAGCTCTGCTGGGGAACAGCAC

CGATCCCGGAGTGTTCACAGATCTGGCCTCCGTGGACAACTCTGAGTTTCAGCAGCTGCTGAATCAGGGCG
TGTCCATGTCTCATAGTACAGCCGAACCAATGCTGATGGAGTACCCCGAAGCCATTACCCGGCTGGTGACCG
GCAGCCAGCGGCCCCCCGACCCCGCTCCAACTCCCCTGGGAACCAGCGGCCTGCCTAATGGGCTGTCCG
GAGATGAAGACTTCTCAAGCATCGCTGATATGGACTTTAGTGCCCTGCTGTCACAGATTTCCTCTAGTGGGTC
TGGCAGCGGCAGCGACCTTTCCCATCCGCCCCCAAGGGGCCATCTGGATGAGCTGACAACCACACTTGAG
TCCATGACCGAGGATCTGAACCTGGACTCACCCCTGACCCCGGAATTGAACGAGATTCTGGATACCTTCCTG
AACGACGAGTGCCTCTTGCATGCCATGCATATCAGCACAGGACTGTCCATCTTCGACACATCTCTGTTTAAGT
AA

- dHF-VPR: pCAG-dAsCpf1-HF(E174R/N282A/S542R/K548R/D908A)-NLS-3xHA-VPR

Human codon optimized Acidaminococcus sp. BV3L6 Cpf1 (AsCpf1) is black, mutant site highlighted with red, nucleoplasmin NLS with blue, 3xHA with orange, VPR with violet

ATGACACAGTTCGAGGGCTTTACCAACCTGTATCAGGTGAGCAAGACACTGCGGTTTGAGCTGATCCCACA
GGGCAAGACCCTGAAGCACATCCAGGAGCAGGGCTTCATCGAGGAGGACAAGGCCCGCAATGATCACTAC
AAGGAGCTGAAGCCCATCATCGATCGGATCTACAAGACCTATGCCGACCAGTGCCTGCAGCTGGTGCAGCT
GGATTGGGAGAACCTGAGCGCCGCCATCGACTCCTATAGAAAGGAGAAAACCGAGGAGACAAGGAACGCC
CTGATCGAGGAGCAGGCCACATATCGCAATGCCATCCACGACTACTTCATCGGCCGGACAGACAACCTGAC
CGATGCCATCAATAAGAGACACGCCGAGATCTACAAGGGCCTGTTCAAGGCCGAGCTGTTTAATGGCAAGGT
GCTGAAGCAGCTGGGCACCGTGACCACAACCGAGCACGAGAACGCCCTGCTGCGGAGCTTCGACAAGTTT
ACAACCTACTTCTCCGGCTTTTATAGAAACAGGAAGAACGTGTTCAGCGCCGAGGACATTAGCACAGCCATC
CCACACCGCATCGTGCAGGACAACTTCCCCAAGTTTAAGGAGAATTGTCACATCTTCACACGCCTGATCACC
GCCGTGCCCAGCCTGCGGGAGCACTTTGAGAACGTGAAGAAGGCCATCGGCATCTTCGTGAGCACCTCCA
TCGAGGAGGTGTTTTCCTTCCCTTTTTATAACCAGCTGCTGACACAGACCCAGATCGACCTGTATAACCAGCT
GCTGGGAGGAATCTCTCGGGAGGCAGGCACCGAGAAGATCAAGGGCCTGAACGAGGTGCTGGCCCTGGC
CATCCAGAAGAATGATGAGACAGCCCACATCATCGCCTCCCTGCCACACAGATTCATCCCCCTGTTTAAGCA
GATCCTGTCCGATAGGAACACCCTGTCTTTCATCCTGGAGGAGTTTAAGAGCGACGAGGAAGTGATCCAGTC
CTTCTGCAAGTACAAGACACTGCTGAGAAACGAGAACGTGCTGGAGACAGCCGAGGCCCTGTTTAACGAGC
TGAACAGCATCGACCTGACACACATCTTCATCAGCCACAAGAAGCTGGAGACAATCAGCAGCGCCCTGTGC
GACCACTGGGATACACTGAGGAATGCCCTGTATGAGCGGAGAATCTCCGAGCTGACAGGCAAGATCACCAA
GTCTGCCAAGGAGAAGGTGCAGCGCAGCCTGAAGCACGAGGATATCAACCTGCAGGAGATCATCTCTGCC
GCAGGCAAGGAGCTGAGCGAGGCCTTCAAGCAGAAAACCAGCGAGATCCTGTCCCACGCACACGCCGCC
CTGGATCAGCCACTGCCTACAACCCTGAAGAAGCAGGAGGAGAAGGAGATCCTGAAGTCTCAGCTGGACA
GCCTGCTGGGCCTGTACCACCTGCTGGACTGGTTTGCCGTGGATGAGTCCAACGAGGTGGACCCCGAGTT
CTCTGCCCGGCTGACCGGCATCAAGCTGGAGATGGAGCCTTCTCTGAGCTTCTACAACAAGGCCAGAAATT
ATGCCACCAAGAAGCCCTACTCCGTGGAGAAGTTCAAGCTGAACTTTCAGATGCCTACACTGGCCAGAGGC
TGGGATGTGAACAGAGAGAAGAACAATGGCGCCATCCTGTTTGTGAAGAACGGCCTGTACTATCTGGGCATC
ATGCCAAAGCAGAAGGGCAGGTATAAGGCCCTGAGCTTCGAGCCCACAGAGAAAACCAGCGAGGGCTTTG
ATAAGATGTACTATGACTACTTCCCTGATGCCGCCAAGATGATCCCAAAGTGCAGCACCCAGCTGAAGGCCG
TGACAGCCCACTTTCAGACCCACACAACCCCCATCCTGCTGTCCAACAATTTCATCGAGCCTCTGGAGATCA
CAAAGGAGATCTACGACCTGAACAATCCTGAGAAGGAGCCAAAGAAGTTTCAGACAGCCTACGCCAAGAAA
ACCGGCGACCAGAAGGGCTACAGAGAGGCCCTGTGCAAGTGGATCGACTTCACAAGGGATTTTCTGTCCAA
GTATACCAAGACAACCTCTATCGATCTGTCTAGCCTGCGGCCATCCTCTCAGTATAAGGACCTGGGCGAGTAC
TATGCCGAGCTGAATCCCCTGCTGTACCACATCAGCTTCCAGAGAATCGCCGAGAAGGAGATCATGGATGCC
GTGGAGACAGGCAAGCTGTACCTGTTCCAGATCTATAACAAGGACTTTGCCAAGGGCCACCACGGCAAGCC
TAATCTGCACACACTGTATTGGACCGGCCTGTTTTCTCCAGAGAACCTGGCCAAGACAAGCATCAAGCTGAA
TGGCCAGGCCGAGCTGTTCTACCGCCCTAAGTCCAGGATGAAGAGGATGGCACACCGGCTGGGAGAGAAG
ATGCTGAACAAGAAGCTGAAGGATCAGAAAACCCCAATCCCCGACACCCTGTACCAGGAGCTGTACGACTAT
GTGAATCACAGACTGTCCCACGACCTGTCTGATGAGGCCAGGGCCCTGCTGCCCAACGTGATCACCAAGGA
GGTGTCTCACGAGATCATCAAGGATAGGCGCTTTACCAGCGACAAGTTCTTTTTCCACGTGCCTATCACACT
GAACTATCAGGCCGCCAATTCCCCATCTAAGTTCAACCAGAGGGTGAATGCCTACCTGAAGGAGCACCCCG

AGACACCTATCATCGGCATCGCCCGGGGCGAGAGAAACCTGATCTATATCACAGTGATCGACTCCACCGGCA
AGATCCTGGAGCAGCGGAGCCTGAACACCATCCAGCAGTTTGATTACCAGAAGAAGCTGGACAACAGGGAG
AAGGAGAGGGTGGCAGCAAGGCAGGCCTGGTCTGTGGTGGGCACAATCAAGGATCTGAAGCAGGGCTATC
TGAGCCAGGTCATCCACGAGATCGTGGACCTGATGATCCACTACCAGGCCGTGGTGGTGCTGGAGAACCTG
AATTTCGGCTTTAAGAGCAAGAGGACCGGCATCGCCGAGAAGGCCGTGTACCAGCAGTTCGAGAAGATGCT
GATCGATAAGCTGAATTGCCTGGTGCTGAAGGACTATCCAGCAGAGAAAGTGGGAGGCGTGCTGAACCCAT
ACCAGCTGACAGACCAGTTCACCTCCTTTGCCAAGATGGGCACCCAGTCTGGCTTCCTGTTTTACGTGCCT
GCCCCATATACATCTAAGATCGATCCCCTGACCGGCTTCGTGGACCCCTTCGTGTGGAAAACCATCAAGAAT
CACGAGAGCCGCAAGCACTTCCTGGAGGGCTTCGACTTTCTGCACTACGACGTGAAAACCGGCGACTTCAT
CCTGCACTTTAAGATGAACAGAAATCTGTCCTTCCAGAGGGGCCTGCCCGGCTTTATGCCTGCATGGGATAT
CGTGTTCGAGAAGAACGAGACACAGTTTGACGCCAAGGGCACCCCTTTCATCGCCGGCAAGAGAATCGTG
CCAGTGATCGAGAATCACAGATTCACCGGCAGATACCGGGACCTGTATCCTGCCAACGAGCTGATCGCCCT
GCTGGAGGAGAAGGGCATCGTGTTCAGGGATGGCTCCAACATCCTGCCAAAGCTGCTGGAGAATGACGATT
CTCACGCCATCGACACCATGGTGGCCCTGATCCGCAGCGTGCTGCAGATGCGGAACTCCAATGCCGCCAC
AGGCGAGGACTATATCAACAGCCCCGTGCGCGATCTGAATGGCGTGTGCTTCGACTCCCGGTTTCAGAACC
CAGAGTGGCCCATGGACGCCGATGCCAATGGCGCCTACCACATCGCCCTGAAGGGCCAGCTGCTGCTGAA
TCACCTGAAGGAGAGCAAGGATCTGAAGCTGCAGAACGGCATCTCCAATCAGGACTGGCTGGCCTACATCC
AGGAGCTGCGCAACAAAAGGCCGGCGGCCACGAAAAAGGCCGGCCAGGCAAAAAAGAAAAAGGGATCCTA
CCCATACGATGTTCCAGATTACGCTTATCCCTACGACGTGCCTGATTATGCATACCCATACGATGTCCCCGACT
ATGCCCTCGAGAGCACCGGTGATGCTTTAGACGATTTTGACTTAGATATGCTTGGTTCAGACGCGTTAGACG
ACTTCGACCTAGACATGTTAGGCTCAGATGCATTGGACGACTTCGATTTAGATATGTTGGGCTCCGATGCCCT
AGATGACTTTGATCTAGATATGCTAGGAGGAAGCGGAGGAGGAGGTAGCGGACCTAAGAAAAAGAGGAAGG
TGGCGGCCGCTGGATCCCCTTCAGGGCAGATCAGCAACCAGGCCCTGGCTCTGGCCCCTAGCTCCGCTCC
AGTGCTGGCCCAGACTATGGTGCCCTCTAGTGCTATGGTGCCTCTGGCCCAGCCACCTGCTCCAGCCCCTG
TGCTGACCCCAGGACCACCCCAGTCACTGAGCGCTCCAGTGCCCAAGTCTACACAGGCCGGCGAGGGGA
CTCTGAGTGAAGCTCTGCTGCACCTGCAGTTCGACGCTGATGAGGACCTGGGAGCTCTGCTGGGGAACAG
CACCGATCCCGGAGTGTTCACAGATCTGGCCTCCGTGGACAACTCTGAGTTTCAGCAGCTGCTGAATCAGG
GCGTGTCCATGTCTCATAGTACAGCCGAACCAATGCTGATGGAGTACCCCGAAGCCATTACCCGGCTGGTGA
CCGGCAGCCAGCGGCCCCCCGACCCCGCTCCAACTCCCCTGGGAACCAGCGGCCTGCCTAATGGGCTGT
CCGGAGATGAAGACTTCTCAAGCATCGCTGATATGGACTTTAGTGCCCTGCTGTCACAGATTTCCTCTAGTG
GGTCTGGCAGCGGCAGCGACCTTTCCCATCCGCCCCCAAGGGGCCATCTGGATGAGCTGACAACCACACT
TGAGTCCATGACCGAGGATCTGAACCTGGACTCACCCCTGACCCCGGAATTGAACGAGATTCTGGATACCTT
CCTGAACGACGAGTGCCTCTTGCATGCCATGCATATCAGCACAGGACTGTCCATCTTCGACACATCTCTGTT
TAAGTAA

- WT-VPR: pCAG-AsCpf1-WT-NLS-3xHA-VPR

Human codon optimized Acidaminococcus sp. BV3L6 Cpf1 (AsCpf1) is black, nucleoplasmin NLS with blue, 3xHA with orange, VPR with violet

ATGACACAGTTCGAGGGCTTTACCAACCTGTATCAGGTGAGCAAGACACTGCGGTTTGAGCTGATCCCACA
GGGCAAGACCCTGAAGCACATCCAGGAGCAGGGCTTCATCGAGGAGGACAAGGCCCGCAATGATCACTAC
AAGGAGCTGAAGCCCATCATCGATCGGATCTACAAGACCTATGCCGACCAGTGCCTGCAGCTGGTGCAGCT
GGATTGGGAGAACCTGAGCGCCGCCATCGACTCCTATAGAAAGGAGAAAACCGAGGAGACAAGGAACGCC
CTGATCGAGGAGCAGGCCACATATCGCAATGCCATCCACGACTACTTCATCGGCCGGACAGACAACCTGAC
CGATGCCATCAATAAGAGACACGCCGAGATCTACAAGGGCCTGTTCAAGGCCGAGCTGTTTAATGGCAAGGT
GCTGAAGCAGCTGGGCACCGTGACCACAACCGAGCACGAGAACGCCCTGCTGCGGAGCTTCGACAAGTTT
ACAACCTACTTCTCCGGCTTTTATGAGAACAGGAAGAACGTGTTCAGCGCCGAGGATATCAGCACAGCCATC
CCACACCGCATCGTGCAGGACAACTTCCCCAAGTTTAAGGAGAATTGTCACATCTTCACACGCCTGATCACC
GCCGTGCCCAGCCTGCGGGAGCACTTTGAGAACGTGAAGAAGGCCATCGGCATCTTCGTGAGCACCTCCA
TCGAGGAGGTGTTTTCCTTCCCTTTTTATAACCAGCTGCTGACACAGACCCAGATCGACCTGTATAACCAGCT
GCTGGGAGGAATCTCTCGGGAGGCAGGCACCGAGAAGATCAAGGGCCTGAACGAGGTGCTGAATCTGGCC

ATCCAGAAGAATGATGAGACAGCCCACATCATCGCCTCCCTGCCACACAGATTCATCCCCCTGTTTAAGCAG
ATCCTGTCCGATAGGAACACCCTGTCTTTCATCCTGGAGGAGTTTAAGAGCGACGAGGAAGTGATCCAGTCC
TTCTGCAAGTACAAGACACTGCTGAGAAACGAGAACGTGCTGGAGACAGCCGAGGCCCTGTTTAACGAGCT
GAACAGCATCGACCTGACACACATCTTCATCAGCCACAAGAAGCTGGAGACAATCAGCAGCGCCCTGTGCG
ACCACTGGGATACACTGAGGAATGCCCTGTATGAGCGGAGAATCTCCGAGCTGACAGGCAAGATCACCAAG
TCTGCCAAGGAGAAGGTGCAGCGCAGCCTGAAGCACGAGGATATCAACCTGCAGGAGATCATCTCTGCCGC
AGGCAAGGAGCTGAGCGAGGCCTTCAAGCAGAAAACCAGCGAGATCCTGTCCCACGCACACGCCGCCCTG
GATCAGCCACTGCCTACAACCCTGAAGAAGCAGGAGGAGAAGGAGATCCTGAAGTCTCAGCTGGACAGCCT
GCTGGGCCTGTACCACCTGCTGGACTGGTTTGCCGTGGATGAGTCCAACGAGGTGGACCCCGAGTTCTCT
GCCCGGCTGACCGGCATCAAGCTGGAGATGGAGCCTTCTCTGAGCTTCTACAACAAGGCCAGAAATTATGC
CACCAAGAAGCCCTACTCCGTGGAGAAGTTCAAGCTGAACTTTCAGATGCCTACACTGGCCTCTGGCTGGG
ACGTGAATAAGGAGAAGAACAATGGCGCCATCCTGTTTGTGAAGAACGGCCTGTACTATCTGGGCATCATGC
CAAAGCAGAAGGGCAGGTATAAGGCCCTGAGCTTCGAGCCCACAGAGAAAACCAGCGAGGGCTTTGATAAG
ATGTACTATGACTACTTCCCTGATGCCGCCAAGATGATCCCAAAGTGCAGCACCCAGCTGAAGGCCGTGACA
GCCCACTTTCAGACCCACACAACCCCCATCCTGCTGTCCAACAATTTCATCGAGCCTCTGGAGATCACAAAG
GAGATCTACGACCTGAACAATCCTGAGAAGGAGCCAAAGAAGTTTCAGACAGCCTACGCCAAGAAAACCGG
CGACCAGAAGGGCTACAGAGAGGCCCTGTGCAAGTGGATCGACTTCACAAGGGATTTTCTGTCCAAGTATA
CCAAGACAACCTCTATCGATCTGTCTAGCCTGCGGCCATCCTCTCAGTATAAGGACCTGGGCGAGTACTATG
CCGAGCTGAATCCCCTGCTGTACCACATCAGCTTCCAGAGAATCGCCGAGAAGGAGATCATGGATGCCGTG
GAGACAGGCAAGCTGTACCTGTTCCAGATCTATAACAAGGACTTTGCCAAGGGCCACCACGGCAAGCCTAAT
CTGCACACACTGTATTGGACCGGCCTGTTTTCTCCAGAGAACCTGGCCAAGACAAGCATCAAGCTGAATGG
CCAGGCCGAGCTGTTCTACCGCCCTAAGTCCAGGATGAAGAGGATGGCACACCGGCTGGGAGAGAAGATG
CTGAACAAGAAGCTGAAGGATCAGAAAACCCCAATCCCCGACACCCTGTACCAGGAGCTGTACGACTATGT
GAATCACAGACTGTCCCACGACCTGTCTGATGAGGCCAGGGCCCTGCTGCCCAACGTGATCACCAAGGAG
GTGTCTCACGAGATCATCAAGGATAGGCGCTTTACCAGCGACAAGTTCTTTTTCCACGTGCCTATCACACTGA
ACTATCAGGCCGCCAATTCCCCATCTAAGTTCAACCAGAGGGTGAATGCCTACCTGAAGGAGCACCCCGAG
ACACCTATCATCGGCATCGATCGGGGCGAGAGAAACCTGATCTATATCACAGTGATCGACTCCACCGGCAAG
ATCCTGGAGCAGCGGAGCCTGAACACCATCCAGCAGTTTGATTACCAGAAGAAGCTGGACAACAGGGAGAA
GGAGAGGGTGGCAGCAAGGCAGGCCTGGTCTGTGGTGGGCACAATCAAGGATCTGAAGCAGGGCTATCTG
AGCCAGGTCATCCACGAGATCGTGGACCTGATGATCCACTACCAGGCCGTGGTGGTGCTGGAGAACCTGAA
TTTCGGCTTTAAGAGCAAGAGGACCGGCATCGCCGAGAAGGCCGTGTACCAGCAGTTCGAGAAGATGCTGA
TCGATAAGCTGAATTGCCTGGTGCTGAAGGACTATCCAGCAGAGAAAGTGGGAGGCGTGCTGAACCCATAC
CAGCTGACAGACCAGTTCACCTCCTTTGCCAAGATGGGCACCCAGTCTGGCTTCCTGTTTTACGTGCCTGC
CCCATATACATCTAAGATCGATCCCCTGACCGGCTTCGTGGACCCCTTCGTGTGGAAAACCATCAAGAATCAC
GAGAGCCGCAAGCACTTCCTGGAGGGCTTCGACTTTCTGCACTACGACGTGAAAACCGGCGACTTCATCCT
GCACTTTAAGATGAACAGAAATCTGTCCTTCCAGAGGGGCCTGCCCGGCTTTATGCCTGCATGGGATATCGT
GTTCGAGAAGAACGAGACACAGTTTGACGCCAAGGGCACCCCTTTCATCGCCGGCAAGAGAATCGTGCCA
GTGATCGAGAATCACAGATTCACCGGCAGATACCGGGACCTGTATCCTGCCAACGAGCTGATCGCCCTGCT
GGAGGAGAAGGGCATCGTGTTCAGGGATGGCTCCAACATCCTGCCAAAGCTGCTGGAGAATGACGATTCTC
ACGCCATCGACACCATGGTGGCCCTGATCCGCAGCGTGCTGCAGATGCGGAACTCCAATGCCGCCACAGG
CGAGGACTATATCAACAGCCCCGTGCGCGATCTGAATGGCGTGTGCTTCGACTCCCGGTTTCAGAACCCAG
AGTGGCCCATGGACGCCGATGCCAATGGCGCCTACCACATCGCCCTGAAGGGCCAGCTGCTGCTGAATCA
CCTGAAGGAGAGCAAGGATCTGAAGCTGCAGAACGGCATCTCCAATCAGGACTGGCTGGCCTACATCCAGG
AGCTGCGCAACAAAAGGCCGGCGGCCACGAAAAAGGCCGGCCAGGCAAAAAAGAAAAAGGGATCCTACCC
ATACGATGTTCCAGATTACGCTTATCCCTACGACGTGCCTGATTATGCATACCCATACGATGTCCCCGACTATG
CCCTCGAGAGCACCGGTGATGCTTTAGACGATTTTGACTTAGATATGCTTGGTTCAGACGCGTTAGACGACT
TCGACCTAGACATGTTAGGCTCAGATGCATTGGACGACTTCGATTTAGATATGTTGGGCTCCGATGCCCTAGA
TGACTTTGATCTAGATATGCTAGGAGGAAGCGGAGGAGGAGGTAGCGGACCTAAGAAAAAGAGGAAGGTGG
CGGCCGCTGGATCCCCTTCAGGGCAGATCAGCAACCAGGCCCTGGCTCTGGCCCCTAGCTCCGCTCCAGT

GCTGGCCCAGACTATGGTGCCCTCTAGTGCTATGGTGCCTCTGGCCCAGCCACCTGCTCCAGCCCCTGTGC
TGACCCCAGGACCACCCCAGTCACTGAGCGCTCCAGTGCCCAAGTCTACACAGGCCGGCGAGGGGACTCT
GAGTGAAGCTCTGCTGCACCTGCAGTTCGACGCTGATGAGGACCTGGGAGCTCTGCTGGGGAACAGCACC
GATCCCGGAGTGTTCACAGATCTGGCCTCCGTGGACAACTCTGAGTTTCAGCAGCTGCTGAATCAGGGCGT
GTCCATGTCTCATAGTACAGCCGAACCAATGCTGATGGAGTACCCCGAAGCCATTACCCGGCTGGTGACCG
GCAGCCAGCGGCCCCCGACCCCGCTCCAACTCCCCTGGGAACCAGCGGCCTGCCTAATGGGCTGTCCG
GAGATGAAGACTTCTCAAGCATCGCTGATATGGACTTTAGTGCCCTGCTGTCACAGATTTCCTCTAGTGGGTC
TGGCAGCGGCAGCGACCTTTCCCATCCGCCCCCAAGGGGCCATCTGGATGAGCTGACAACCACACTTGAG
TCCATGACCGAGGATCTGAACCTGGACTCACCCCTGACCCCGGAATTGAACGAGATTCTGGATACCTTCCTG
AACGACGAGTGCCTCTTGCATGCCATGCATATCAGCACAGGACTGTCCATCTTCGACACATCTCTGTTTAAGT
AA

- dRKA-VPR: pCAG-dAsCpf1-RKA(E174R/R951K/R955A/D908A)-NLS-3xHA-VPR

Human codon optimized Acidaminococcus sp. BV3L6 Cpf1 (AsCpf1) is black, mutant site highlighted with red, nucleoplasmin NLS with blue, 3xHA with orange, VPR with violet

ATGACACAGTTCGAGGGCTTTACCAACCTGTATCAGGTGAGCAAGACACTGCGGTTTGAGCTGATCCCACA
GGGCAAGACCCTGAAGCACATCCAGGAGCAGGGCTTCATCGAGGAGGACAAGGCCCGCAATGATCACTAC
AAGGAGCTGAAGCCCATCATCGATCGGATCTACAAGACCTATGCCGACCAGTGCCTGCAGCTGGTGCAGCT
GGATTGGGAGAACCTGAGCGCCGCCATCGACTCCTATAGAAAGGAGAAAACCGAGGAGACAAGGAACGCC
CTGATCGAGGAGCAGGCCACATATCGCAATGCCATCCACGACTACTTCATCGGCCGGACAGACAACCTGAC
CGATGCCATCAATAAGAGACACGCCGAGATCTACAAGGGCCTGTTCAAGGCCGAGCTGTTTAATGGCAAGGT
GCTGAAGCAGCTGGGCACCGTGACCACAACCGAGCACGAGAACGCCCTGCTGCGGAGCTTCGACAAGTTT
ACAACCTACTTCTCCGGCTTTTATAGAAACAGGAAGAACGTGTTCAGCGCCGAGGACATTAGCACAGCCATC
CCACACCGCATCGTGCAGGACAACTTCCCCAAGTTTAAGGAGAATTGTCACATCTTCACACGCCTGATCACC
GCCGTGCCCAGCCTGCGGGAGCACTTTGAGAACGTGAAGAAGGCCATCGGCATCTTCGTGAGCACCTCCA
TCGAGGAGGTGTTTTCCTTCCCTTTTTATAACCAGCTGCTGACACAGACCCAGATCGACCTGTATAACCAGCT
GCTGGGAGGAATCTCTCGGGAGGCAGGCACCGAGAAGATCAAGGGCCTGAACGAGGTGCTGAATCTGGCC
ATCCAGAAGAATGATGAGACAGCCCACATCATCGCCTCCCTGCCACACAGATTCATCCCCCTGTTTAAGCAG
ATCCTGTCCGATAGGAACACCCTGTCTTTCATCCTGGAGGAGTTTAAGAGCGACGAGGAAGTGATCCAGTCC
TTCTGCAAGTACAAGACACTGCTGAGAAACGAGAACGTGCTGGAGACAGCCGAGGCCCTGTTTAACGAGCT
GAACAGCATCGACCTGACACACATCTTCATCAGCCACAAGAAGCTGGAGACAATCAGCAGCGCCCTGTGCG
ACCACTGGGATACACTGAGGAATGCCCTGTATGAGCGGAGAATCTCCGAGCTGACAGGCAAGATCACCAAG
TCTGCCAAGGAGAAGGTGCAGCGCAGCCTGAAGCACGAGGATATCAACCTGCAGGAGATCATCTCTGCCGC
AGGCAAGGAGCTGAGCGAGGCCTTCAAGCAGAAAACCAGCGAGATCCTGTCCCACGCACACGCCGCCCTG
GATCAGCCACTGCCTACAACCCTGAAGAAGCAGGAGGAGAAGGAGATCCTGAAGTCTCAGCTGGACAGCCT
GCTGGGCCTGTACCACCTGCTGGACTGGTTTGCCGTGGATGAGTCCAACGAGGTGGACCCCGAGTTCTCT
GCCCGGCTGACCGGCATCAAGCTGGAGATGGAGCCTTCTCTGAGCTTCTACAACAAGGCCAGAAATTATGC
CACCAAGAAGCCCTACTCCGTGGAGAAGTTCAAGCTGAACTTTCAGATGCCTACACTGGCCTCTGGCTGGG
ACGTGAATAAGGAGAAGAACAATGGCGCCATCCTGTTTGTGAAGAACGGCCTGTACTATCTGGGCATCATGC
CAAAGCAGAAGGGCAGGTATAAGGCCCTGAGCTTCGAGCCCACAGAGAAAACCAGCGAGGGCTTTGATAAG
ATGTACTATGACTACTTCCCTGATGCCGCCAAGATGATCCCAAAGTGCAGCACCCAGCTGAAGGCCGTGACA
GCCCACTTTCAGACCCACACAACCCCCATCCTGCTGTCCAACAATTTCATCGAGCCTCTGGAGATCACAAAG
GAGATCTACGACCTGAACAATCCTGAGAAGGAGCCAAAGAAGTTTCAGACAGCCTACGCCAAGAAAACCGG
CGACCAGAAGGGCTACAGAGAGGCCCTGTGCAAGTGGATCGACTTCACAAGGGATTTTCTGTCCAAGTATA
CCAAGACAACCTCTATCGATCTGTCTAGCCTGCGGCCATCCTCTCAGTATAAGGACCTGGGCGAGTACTATG
CCGAGCTGAATCCCCTGCTGTACCACATCAGCTTCCAGAGAATCGCCGAGAAGGAGATCATGGATGCCGTG
GAGACAGGCAAGCTGTACCTGTTCCAGATCTATAACAAGGACTTTGCCAAGGGCCACCACGGCAAGCCTAAT
CTGCACACACTGTATTGGACCGGCCTGTTTTCTCCAGAGAACCTGGCCAAGACAAGCATCAAGCTGAATGG
CCAGGCCGAGCTGTTCTACCGCCCTAAGTCCAGGATGAAGAGGATGGCACACCGGCTGGGAGAGAAGATG
CTGAACAAGAAGCTGAAGGATCAGAAAACCCCAATCCCCGACACCCTGTACCAGGAGCTGTACGACTATGT

GAATCACAGACTGTCCCACGACCTGTCTGATGAGGCCAGGGCCCTGCTGCCCAACGTGATCACCAAGGAG
GTGTCTCACGAGATCATCAAGGATAGGCGCTTTACCAGCGACAAGTTCTTTTTCCACGTGCCTATCACACTGA
ACTATCAGGCCGCCAATTCCCCATCTAAGTTCAACCAGAGGGTGAATGCCTACCTGAAGGAGCACCCCGAG
ACACCTATCATCGGCATCGATCGGGGCGAGAGAAACCTGATCTATATCACAGTGATCGACTCCACCGGCAAG
ATCCTGGAGCAGCGGAGCCTGAACACCATCCAGCAGTTTGATTACCAGAAGAAGCTGGACAACAGGGAGAA
GGAGAAGGTGGCGGCCGCTCAGGCCTGGTCTGTGGTGGGCACAATCAAGGATCTGAAGCAGGGCTATCTG
AGCCAGGTCATCCACGAGATCGTGGACCTGATGATCCACTACCAGGCCGTGGTGGTGCTGGAGAACCTGAA
TTTCGGCTTTAAGAGCAAGAGGACCGGCATCGCCGAGAAGGCCGTGTACCAGCAGTTCGAGAAGATGCTGA
TCGATAAGCTGAATTGCCTGGTGCTGAAGGACTATCCAGCAGAGAAAGTGGGAGGCGTGCTGAACCCATAC
CAGCTGACAGACCAGTTCACCTCCTTTGCCAAGATGGGCACCCAGTCTGGCTTCCTGTTTTACGTGCCTGC
CCCATATACATCTAAGATCGATCCCCTGACCGGCTTCGTGGACCCCTTCGTGTGGAAAACCATCAAGAATCAC
GAGAGCCGCAAGCACTTCCTGGAGGGCTTCGACTTTCTGCACTACGACGTGAAAACCGGCGACTTCATCCT
GCACTTTAAGATGAACAGAAATCTGTCCTTCCAGAGGGGCCTGCCCGGCTTTATGCCTGCATGGGATATCGT
GTTCGAGAAGAACGAGACACAGTTTGACGCCAAGGGCACCCCTTTCATCGCCGGCAAGAGAATCGTGCCA
GTGATCGAGAATCACAGATTCACCGGCAGATACCGGGACCTGTATCCTGCCAACGAGCTGATCGCCCTGCT
GGAGGAGAAGGGCATCGTGTTCAGGGATGGCTCCAACATCCTGCCAAAGCTGCTGGAGAATGACGATTCTC
ACGCCATCGACACCATGGTGGCCCTGATCCGCAGCGTGCTGCAGATGCGGAACTCCAATGCCGCCACAGG
CGAGGACTATATCAACAGCCCCGTGCGCGATCTGAATGGCGTGTGCTTCGACTCCCGGTTTCAGAACCCAG
AGTGGCCCATGGACGCCGATGCCAATGGCGCCTACCACATCGCCCTGAAGGGCCAGCTGCTGCTGAATCA
CCTGAAGGAGAGCAAGGATCTGAAGCTGCAGAACGGCATCTCCAATCAGGACTGGCTGGCCTACATCCAGG
AGCTGCGCAACAAAAAGGCCGGCGGCCACGAAAAAGGCCGGCCAGGCAAAAAAGAAAAAGGGATCCTACC
CATACGATGTTCCAGATTACGCTTATCCCTACGACGTGCCTGATTATGCATACCCATACGATGTCCCCGACTAT
GCCCTCGAGAGCACCGGTGATGCTTTAGACGATTTTGACTTAGATATGCTTGGTTCAGACGCGTTAGACGAC
TTCGACCTAGACATGTTAGGCTCAGATGCATTGGACGACTTCGATTTAGATATGTTGGGCTCCGATGCCCTAG
ATGACTTTGATCTAGATATGCTAGGAGGAAGCGGAGGAGGAGGTAGCGGACCTAAGAAAAAGAGGAAGGTG
GCGGCCGCTGGATCCCCTTCAGGGCAGATCAGCAACCAGGCCCTGGCTCTGGCCCCTAGCTCCGCTCCAG
TGCTGGCCCAGACTATGGTGCCCTCTAGTGCTATGGTGCCTCTGGCCCAGCCACCTGCTCCAGCCCCTGTG
CTGACCCCAGGACCACCCCAGTCACTGAGCGCTCCAGTGCCCAAGTCTACACAGGCCGGCGAGGGGACTC
TGAGTGAAGCTCTGCTGCACCTGCAGTTCGACGCTGATGAGGACCTGGGAGCTCTGCTGGGGAACAGCAC
CGATCCCGGAGTGTTCACAGATCTGGCCTCCGTGGACAACTCTGAGTTTCAGCAGCTGCTGAATCAGGGCG
TGTCCATGTCTCATAGTACAGCCGAACCAATGCTGATGGAGTACCCCGAAGCCATTACCCGGCTGGTGACCG
GCAGCCAGCGGCCCCCCGACCCCGCTCCAACTCCCCTGGGAACCAGCGGCCTGCCTAATGGGCTGTCCG
GAGATGAAGACTTCTCAAGCATCGCTGATATGGACTTTAGTGCCCTGCTGTCACAGATTTCCTCTAGTGGGTC
TGGCAGCGGCAGCGACCTTTCCCATCCGCCCCCAAGGGGCCATCTGGATGAGCTGACAACCACACTTGAG
TCCATGACCGAGGATCTGAACCTGGACTCACCCCTGACCCCGGAATTGAACGAGATTCTGGATACCTTCCTG
AACGACGAGTGCCTCTTGCATGCCATGCATATCAGCACAGGACTGTCCATCTTCGACACATCTCTGTTTAAGT
AA

- dHF-VPR: pCAG-dAsCpf1-HF(E174R/N282A/S542R/K548R/D908A)-NLS-3xHA-VPR

Human codon optimized Acidaminococcus sp. BV3L6 Cpf1 (AsCpf1) is black, mutant site highlighted with red, nucleoplasmin NLS with blue, 3xHA with orange, VPR with violet

ATGACACAGTTCGAGGGCTTTACCAACCTGTATCAGGTGAGCAAGACACTGCGGTTTGAGCTGATCCCACA
GGGCAAGACCCTGAAGCACATCCAGGAGCAGGGCTTCATCGAGGAGGACAAGGCCCGCAATGATCACTAC
AAGGAGCTGAAGCCCATCATCGATCGGATCTACAAGACCTATGCCGACCAGTGCCTGCAGCTGGTGCAGCT
GGATTGGGAGAACCTGAGCGCCGCCATCGACTCCTATAGAAAGGAGAAAACCGAGGAGACAAGGAACGCC
CTGATCGAGGAGCAGGCCACATATCGCAATGCCATCCACGACTACTTCATCGGCCGGACAGACAACCTGAC
CGATGCCATCAATAAGAGACACGCCGAGATCTACAAGGGCCTGTTCAAGGCCGAGCTGTTTAATGGCAAGGT
GCTGAAGCAGCTGGGCACCGTGACCACAACCGAGCACGAGAACGCCCTGCTGCGGAGCTTCGACAAGTTT
ACAACCTACTTCTCCGGCTTTTATAGAAACAGGAAGAACGTGTTCAGCGCCGAGGACATTAGCACAGCCATC

CCACACCGCATCGTGCAGGACAACTTCCCCAAGTTTAAGGAGAATTGTCACATCTTCACACGCCTGATCACC
GCCGTGCCCAGCCTGCGGGAGCACTTTGAGAACGTGAAGAAGGCCATCGGCATCTTCGTGAGCACCTCCA
TCGAGGAGGTGTTTTCCTTCCCTTTTTATAACCAGCTGCTGACACAGACCCAGATCGACCTGTATAACCAGCT
GCTGGGAGGAATCTCTCGGGAGGCAGGCACCGAGAAGATCAAGGGCCTGAACGAGGTGCTGGCCCTGGC
CATCCAGAAGAATGATGAGACAGCCCACATCATCGCCTCCCTGCCACACAGATTCATCCCCCTGTTTAAGCA
GATCCTGTCCGATAGGAACACCCTGTCTTTCATCCTGGAGGAGTTTAAGAGCGACGAGGAAGTGATCCAGTC
CTTCTGCAAGTACAAGACACTGCTGAGAAACGAGAACGTGCTGGAGACAGCCGAGGCCCTGTTTAACGAGC
TGAACAGCATCGACCTGACACACATCTTCATCAGCCACAAGAAGCTGGAGACAATCAGCAGCGCCCTGTGC
GACCACTGGGATACACTGAGGAATGCCCTGTATGAGCGGAGAATCTCCGAGCTGACAGGCAAGATCACCAA
GTCTGCCAAGGAGAAGGTGCAGCGCAGCCTGAAGCACGAGGATATCAACCTGCAGGAGATCATCTCTGCC
GCAGGCAAGGAGCTGAGCGAGGCCTTCAAGCAGAAAACCAGCGAGATCCTGTCCCACGCACACGCCGCC
CTGGATCAGCCACTGCCTACAACCCTGAAGAAGCAGGAGGAGAAGGAGATCCTGAAGTCTCAGCTGGACA
GCCTGCTGGGCCTGTACCACCTGCTGGACTGGTTTGCCGTGGATGAGTCCAACGAGGTGGACCCCGAGTT
CTCTGCCCGGCTGACCGGCATCAAGCTGGAGATGGAGCCTTCTCTGAGCTTCTACAACAAGGCCAGAAATT
ATGCCACCAAGAAGCCCTACTCCGTGGAGAAGTTCAAGCTGAACTTTCAGATGCCTACACTGGCCAGAGGC
TGGGATGTGAACAGAGAGAAGAACAATGGCGCCATCCTGTTTGTGAAGAACGGCCTGTACTATCTGGGCATC
ATGCCAAAGCAGAAGGGCAGGTATAAGGCCCTGAGCTTCGAGCCCACAGAGAAAACCAGCGAGGGCTTTG
ATAAGATGTACTATGACTACTTCCCTGATGCCGCCAAGATGATCCCAAAGTGCAGCACCCAGCTGAAGGCCG
TGACAGCCCACTTTCAGACCCACACAACCCCCATCCTGCTGTCCAACAATTTCATCGAGCCTCTGGAGATCA
CAAAGGAGATCTACGACCTGAACAATCCTGAGAAGGAGCCAAAGAAGTTTCAGACAGCCTACGCCAAGAAA
ACCGGCGACCAGAAGGGCTACAGAGAGGCCCTGTGCAAGTGGATCGACTTCACAAGGGATTTTCTGTCCAA
GTATACCAAGACAACCTCTATCGATCTGTCTAGCCTGCGGCCATCCTCTCAGTATAAGGACCTGGGCGAGTAC
TATGCCGAGCTGAATCCCCTGCTGTACCACATCAGCTTCCAGAGAATCGCCGAGAAGGAGATCATGGATGCC
GTGGAGACAGGCAAGCTGTACCTGTTCCAGATCTATAACAAGGACTTTGCCAAGGGCCACCACGGCAAGCC
TAATCTGCACACACTGTATTGGACCGGCCTGTTTTCTCCAGAGAACCTGGCCAAGACAAGCATCAAGCTGAA
TGGCCAGGCCGAGCTGTTCTACCGCCCTAAGTCCAGGATGAAGAGGATGGCACACCGGCTGGGAGAGAAG
ATGCTGAACAAGAAGCTGAAGGATCAGAAAACCCCAATCCCCGACACCCTGTACCAGGAGCTGTACGACTAT
GTGAATCACAGACTGTCCCACGACCTGTCTGATGAGGCCAGGGCCCTGCTGCCCAACGTGATCACCAAGGA
GGTGTCTCACGAGATCATCAAGGATAGGCGCTTTACCAGCGACAAGTTCTTTTTCCACGTGCCTATCACACT
GAACTATCAGGCCGCCAATTCCCCATCTAAGTTCAACCAGAGGGTGAATGCCTACCTGAAGGAGCACCCCG
AGACACCTATCATCGGCATCGATCGGGGCGAGAGAAACCTGATCTATATCACAGTGATCGACTCCACCGGCA
AGATCCTGGAGCAGCGGAGCCTGAACACCATCCAGCAGTTTGATTACCAGAAGAAGCTGGACAACAGGGAG
AAGGAGAGGGTGGCAGCAAGGCAGGCCTGGTCTGTGGTGGGCACAATCAAGGATCTGAAGCAGGGCTATC
TGAGCCAGGTCATCCACGAGATCGTGGACCTGATGATCCACTACCAGGCCGTGGTGGTGCTGGAGAACCTG
AATTTCGGCTTTAAGAGCAAGAGGACCGGCATCGCCGAGAAGGCCGTGTACCAGCAGTTCGAGAAGATGCT
GATCGATAAGCTGAATTGCCTGGTGCTGAAGGACTATCCAGCAGAGAAAGTGGGAGGCGTGCTGAACCCAT
ACCAGCTGACAGACCAGTTCACCTCCTTTGCCAAGATGGGCACCCAGTCTGGCTTCCTGTTTTACGTGCCT
GCCCCATATACATCTAAGATCGATCCCCTGACCGGCTTCGTGGACCCCTTCGTGTGGAAAACCATCAAGAAT
CACGAGAGCCGCAAGCACTTCCTGGAGGGCTTCGACTTTCTGCACTACGACGTGAAAACCGGCGACTTCAT
CCTGCACTTTAAGATGAACAGAAATCTGTCCTTCCAGAGGGGCCTGCCCGGCTTTATGCCTGCATGGGATAT
CGTGTTCGAGAAGAACGAGACACAGTTTGACGCCAAGGGCACCCCTTTCATCGCCGGCAAGAGAATCGTG
CCAGTGATCGAGAATCACAGATTCACCGGCAGATACCGGGACCTGTATCCTGCCAACGAGCTGATCGCCCT
GCTGGAGGAGAAGGGCATCGTGTTCAGGGATGGCTCCAACATCCTGCCAAAGCTGCTGGAGAATGACGATT
CTCACGCCATCGACACCATGGTGGCCCTGATCCGCAGCGTGCTGCAGATGCGGAACTCCAATGCCGCCAC
AGGCGAGGACTATATCAACAGCCCCGTGCGCGATCTGAATGGCGTGTGCTTCGACTCCCGGTTTCAGAACC
CAGAGTGGCCCATGGACGCCGATGCCAATGGCGCCTACCACATCGCCCTGAAGGGCCAGCTGCTGCTGAA
TCACCTGAAGGAGAGCAAGGATCTGAAGCTGCAGAACGGCATCTCCAATCAGGACTGGCTGGCCTACATCC
AGGAGCTGCGCAACAAAAGGCCGGCGGCCACGAAAAAGGCCGGCCAGGCAAAAAAGAAAAAGGGATCCTA
CCCATACGATGTTCCAGATTACGCTTATCCCTACGACGTGCCTGATTATGCATACCCATACGATGTCCCCGACT

ATGCCCTCGAGAGCACCGGTGATGCTTTAGACGATTTTGACTTAGATATGCTTGGTTCAGACGCGTTAGACG
ACTTCGACCTAGACATGTTAGGCTCAGATGCATTGGACGACTTCGATTTAGATATGTTGGGCTCCGATGCCCT
AGATGACTTTGATCTAGATATGCTAGGAGGAAGCGGAGGAGGAGGTAGCGGACCTAAGAAAAAGAGGAAGG
TGGCGGCCGCTGGATCCCCTTCAGGGCAGATCAGCAACCAGGCCCTGGCTCTGGCCCCTAGCTCCGCTCC
AGTGCTGGCCCAGACTATGGTGCCCTCTAGTGCTATGGTGCCTCTGGCCCAGCCACCTGCTCCAGCCCCTG
TGCTGACCCCAGGACCACCCCAGTCACTGAGCGCTCCAGTGCCCAAGTCTACACAGGCCGGCGAGGGGA
CTCTGAGTGAAGCTCTGCTGCACCTGCAGTTCGACGCTGATGAGGACCTGGGAGCTCTGCTGGGGAACAG
CACCGATCCCGGAGTGTTCACAGATCTGGCCTCCGTGGACAACTCTGAGTTTCAGCAGCTGCTGAATCAGG
GCGTGTCCATGTCTCATAGTACAGCCGAACCAATGCTGATGGAGTACCCCGAAGCCATTACCCGGCTGGTGA
CCGGCAGCCAGCGGCCCCCCGACCCCGCTCCAACTCCCCTGGGAACCAGCGGCCTGCCTAATGGGCTGT
CCGGAGATGAAGACTTCTCAAGCATCGCTGATATGGACTTTAGTGCCCTGCTGTCACAGATTTCCTCTAGTG
GGTCTGGCAGCGGCAGCGACCTTTCCCATCCGCCCCCAAGGGGCCATCTGGATGAGCTGACAACCACACT
TGAGTCCATGACCGAGGATCTGAACCTGGACTCACCCCTGACCCCGGAATTGAACGAGATTCTGGATACCTT
CCTGAACGACGAGTGCCTCTTGCATGCCATGCATATCAGCACAGGACTGTCCATCTTCGACACATCTCTGTT
TAAGTAA

- The amino acid sequences of the *As*Cpf1 variants used in this study

*As*Cpf1-A mutant site is K780A
*As*Cpf1-KA mutant sites are R951K, R955A
*As*Cpf1-AKA mutant sites are K780A, R951K, R955A
*As*Cpf1-RKA (*As*Cpf1-Plus) mutant sites are E174R, R951K, R955A
*As*Cpf1-KK mutant sites are R951K, R955K
*As*Cpf1-AKK mutant sites are K780A, R951K, R955K
*As*Cpf1-RKK mutant sites are E174R, R951K, R955K
en*As*Cpf1-HF mutant sites are E174R, N282A, S542R, K548R
*As*Cpf1-ultra mutant sites are M537R, F870L
*As*Cpf1-UKA mutant sites are M537R, F870L, R951K, R955A
*As*Cpf1-UKK mutant sites are M537R, F870L, R951K, R955K
*As*Cpf1-RU mutant sites are E174R, M537R, F870L
*As*Cpf1-RKAU mutant sites are E174R, M537R, F870L, R951K, R955A
*As*Cpf1-RKKU mutant sites are E174R, M537R, F870L, R951K, R955K
en*As*Cpf1 mutant sites are E174R, S542R, K548R

MTQFEGFTNLYQVSKTLRFELIPQGKTLKHIQEQGFIEEDKARNDHYKELKPIIDRIYKTYADQCLQLVQLDWENL
SAAIDSYRKEKTEETRNALIEEQATYRNAIHDYFIGRTDNLTDAINKRHAEIYKGLFKAELFNGKVLKQLGTVTTTE
HENALLRSFDKFTTYFSGFY**E(174)**NRKNVFSAEDISTAIPHRIVQDNFPKFKENCHIFTRLITAVPSLREHFENVKK
AIGIFVSTSIEEVFSFPFYNQLLTQTQIDLYNQLLGGISREAGTEKIKGLNEVL**N(282)**LAIQKNDETAHIIASLPHRFI
PLFKQILSDRNTLSFILEEFKSDEEVIQSFCKYKTLLRNENVLETAEALFNELNSIDLTHIFISHKKLETISSALCDHW
DTLRNALYERRISELTGKITKSAKEKVQRSLKHEDINLQEIISAAGKELSEAFKQKTSEILSHAHAALDQPLPTTLKK
QEEKEILKSQLDSLLGLYHLLDWFAVDESNEVDPEFSARLTGIKLEMEPSLSFYNKARNYATKKPYSVEKFKLNF
Q**M(537)**PTLA**S(542)**GWDVN**K(548)**EKNNGAILFVKNGLYYLGIMPKQKGRYKALSFEPTEKTSEGFDKMYYDYF
PDAAKMIPKCSTQLKAVTAHFQTHTTPILLSNNFIEPLEITKEIYDLNNPEKEPKKFQTAYAKKTGDQKGYREALCK
WIDFTRDFLSKYTKTTSIDLSSLRPSSQYKDLGEYYAELNPLLYHISFQRIAEKEIMDAVETGKLYLFQIYNKDFAK
GHHGKPNLHTLYWTGLFSPENLAKTSI**K(780)**LNGQAELFYRPKSRMKRMAHRLGEKMLNKKLKDQKTPIPDTLY
QELYDYVNHRLSHDLSDEARALLPNVITKEVSHEIIKDRRFTSDKF**F(870)**FHVPITLNYQAANSPSKFNQRVNAYL
KEHPETPIIGIDRGERNLIYITVIDSTGKILEQRSLNTIQQFDYQKKLDNREKE**R(951)**VAA**R(955)**QAWSVVGTIKDL
KQGYLSQVIHEIVDLMIHYQAVVVLENLNFGFKSKRTGIAEKAVYQQFEKMLIDKLNCLVLKDYPAEKVGGVLNPY
QLTDQFTSFAKMGTQSGFLFYVPAPYTSKIDPLTGFVDPFVWKTIKNHESRKHFLEGFDFLHYDVKTGDFILHFK
MNRNLSFQRGLPGFMPAWDIVFEKNETQFDAKGTPFIAGKRIVPVIENHRFTGRYRDLYPANELIALLEEKGIVFR

DGSNILPKLLENDDSHAIDTMVALIRSVLQMRNSNAATGEDYINSPVRDLNGVCFDSRFQNPEWPMDADANGAY
HIALKGQLLLNHLKESKDLKLQNGISNQDWLAYIQELRN

- The amino acid sequences of the *Lb*Cpf1 variants used in this study:

*Lb*Cpf1-KA mutant sites are R883K, R887A
*Lb*Cpf1-RKA (*Lb*Cpf1-Plus) mutant sites are D156R, R883K, R887A
*Lb*Cpf1-KK mutant sites are R883K, R887K
*Lb*Cpf1-RKK mutant sites are D156R, R883K, R887K
*Lb*Cpf1-ultra mutant sites are N527R, E795L
*Lb*Cpf1-UKA mutant sites are N527R, E795L, R883K, R887A
*Lb*Cpf1-UKK mutant sites are N527R, E795L, R883K, R887K
*Lb*Cpf1-RU mutant sites are D156R, N527R, E795L
*Lb*Cpf1-RKAU mutant sites are D156R, N527R, E795L, R883K, R887A
*Lb*Cpf1-RKKU mutant sites are D156R, N527R, E795L, R883K, R887K

MSKLEKFTNCYSLSKTLRFKAIPVGKTQENIDNKRLLVEDEKRAEDYKGVKKLLDRYYLSFINDVLHSIKLKNLNNY
ISLFRKKTRTEKENKELENLEINLRKEIAKAFKGNEGYKSLFKKDIIETILPEFLDDKDEIALVNSFNGFTTAFTGFF**D(**
156)NRENMFSEEAKSTSIAFRCINENLTRYISNMDIFEKVDAIFDKHEVQEIKEKILNSDYDVEDFFEGEFFNFVLTQ
EGIDVYNAIIGGFVTESGEKIKGLNEYINLYNQKTKQKLPKFKPLYKQVLSDRESLSFYGEGYTSDEEVLEVFRNTL
NKNSEIFSSIKKLEKLFKNFDEYSSAGIFVKNGPAISTISKDIFGEWNVIRDKWNAEYDDIHLKKKAVVTEKYEDDR
RKSFKKIGSFSLEQLQEYADADLSVVEKLKEIIIQKVDEIYKVYGSSEKLFDADFVLEKSLKKNDAVVAIMKDLLDSV
KSFENYIKAFFGEGKETNRDESFYGDFVLAYDILLKVDHIYDAIRNYVTQKPYSKDKFKLYFQ**N(527)**PQFMGGWD
KDKETDYRATILRYGSKYYLAIMDKKYAKCLQKIDKDDVNGNYEKINYKLLPGPNKMLPKVFFSKKWMAYYNPSE
DIQKIYKNGTFKKGDMFNLNDCHKLIDFFKDSISRYPKWSNAYDFNFSETEKYKDIAGFYREVEEQGYKVSFESA
SKKEVDKLVEEGKLYMFQIYNKDFSDKSHGTPNLHTMYFKLLFDENNHGQIRLSGGAELFMRRASLKKEELVVHP
ANSPIANKNPDNPKKTTTLSYDVYKDKRFSEDQY**E(795)**LHIPIAINKCPKNIFKINTEVRVLLKHDDNPYVIGIDRG
ERNLLYIVVVDGKGNIVEQYSLNEIINNFNGIRIKTDYHSLLDKKEKE**R(883)**FEA**R(887)**QNWTSIENIKELKAGYIS
QVVHKICELVEKYDAVIALEDLNSGFKNSRVKVEKQVYQKFEKMLIDKLNYMVDKKSNPCATGGALKGYQITNKF
ESFKSMSTQNGFIFYIPAWLTSKIDPSTGFVNLLKTKYTSIADSKKFISSFDRIMYVPEEDLFEFALDYKNFSRTDAD
YIKKWKLYSYGNRIRIFRNPKKNNVFDWEEVCLTSAYKELFNKYGINYQQGDIRALLCEQSDKAFYSSFMALMSL
MLQMRNSITGRTDVDFLISPVKNSDGIFYDSRNYEAQENAILPKNADANGAYNIARKVLWAIGQFKKAEDEKLDKV
KIAISNKEWLEYAQTSVKH
